# High-Performance Genetically Encoded Green Fluorescent Biosensors for Intracellular l-Lactate

**DOI:** 10.1021/acscentsci.3c01250

**Published:** 2024-01-31

**Authors:** Saaya Hario, Giang N. T. Le, Hikaru Sugimoto, Kei Takahashi-Yamashiro, Suguru Nishinami, Hirofumi Toda, Selene Li, Jonathan S. Marvin, Shinya Kuroda, Mikhail Drobizhev, Takuya Terai, Yusuke Nasu, Robert E. Campbell

**Affiliations:** † Department of Chemistry, Graduate School of Science, The University of Tokyo, Bunkyo-ku, Tokyo 113-0033, Japan; ‡ Department of Chemistry, University of Toronto, Toronto, Ontario M5S 3H6, Canada; § Department of Biochemistry and Molecular Biology, Graduate School of Medicine, The University of Tokyo, Bunkyo-ku, Tokyo 113-0033, Japan; ∥ Department of Molecular Pathology, Graduate School of Medicine, The University of Tokyo, Bunkyo-ku, Tokyo 113-0033, Japan; ⊥ Department of Chemistry, Faculty of Science, University of Alberta, Edmonton, Alberta T6G 2G2, Canada; # International Institute for Integrative Sleep Medicine, University of Tsukuba, Tsukuba, Ibaraki 305-8575, Japan; ∇ Howard Hughes Medical Institute, Janelia Research Campus, Ashburn, Virginia 20147, United States; • Department of Biological Sciences, Graduate School of Science, University of Tokyo, Bunkyo-ku, Tokyo 113-0033, Japan; ° Department of Microbiology and Cell Biology, Montana State University, Bozeman, Montana 59717, United States; ¶ PRESTO, Japan Science and Technology Agency, Chiyoda-ku, Tokyo 102-0075, Japan; □ CERVO Brain Research Center and Department of Biochemistry, Microbiology, and Bioinformatics, Université Laval, Québec, Québec G1 V 0A6, Canada

## Abstract

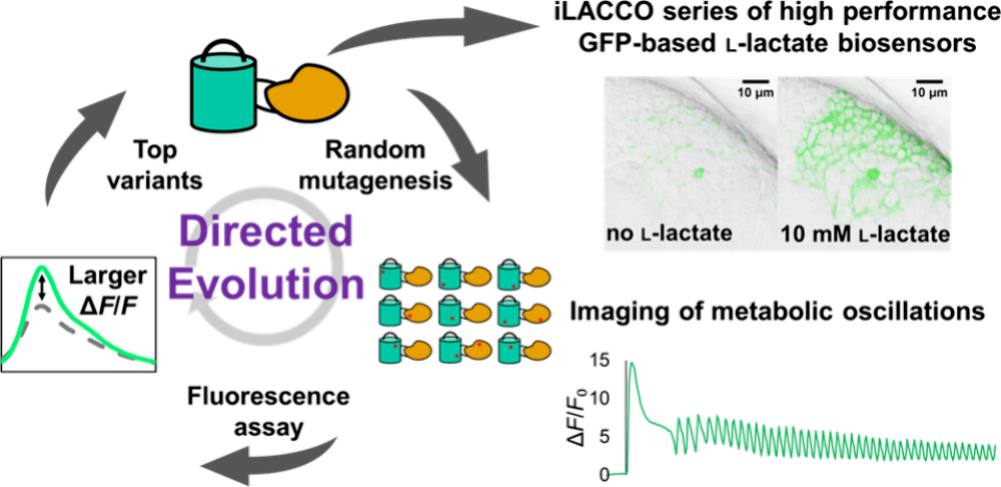

l-Lactate is a monocarboxylate produced during the process of cellular glycolysis and has long generally been considered a waste product. However, studies in recent decades have provided new perspectives on the physiological roles of l-lactate as a major energy substrate and a signaling molecule. To enable further investigations of the physiological roles of l-lactate, we have developed a series of high-performance (Δ*F*/*F* = 15 to 30 *in vitro*), intensiometric, genetically encoded green fluorescent protein (GFP)-based intracellular l-lactate biosensors with a range of affinities. We evaluated these biosensors in cultured cells and demonstrated their application in an *ex vivo* preparation of *Drosophila* brain tissue. Using these biosensors, we were able to detect glycolytic oscillations, which we analyzed and mathematically modeled.

## Abstract 2

We engineered high-performance green fluorescent protein-based l-lactate biosensors and demonstrated their utility in applications such as metabolic oscillation imaging.

## Introduction

Lactate is a monocarboxylate produced from pyruvate by lactate dehydrogenase (LDH) during the process of glycolysis. It can exist as two stereoisomers, l-lactate and d-lactate, with the former being the predominant enantiomer in the human body.^1^l-Lactate has long had a reputation as a metabolic waste product, supported by reports such as a 1929 study that revealed a strong correlation between l-lactate concentration and muscle fatigue in frogs.^2^ However, more recent studies have revealed that this unfavorable reputation is undeserved and should be reconsidered.^3^l-Lactate is now known to have many favorable physiological roles as both an energy fuel source^4^ and a signaling molecule,^5,6^ in processes that include memory consolidation,^7^ immune response,^8,9^ and neurogenesis.^10^ That said, l-lactate is also unfavorably implicated in a number of pathological processes, including inflammation,^11,12^ cancer,^13−16^ and neurodegeneration.^17,18^

Growing evidence that l-lactate has a variety of both favorable and unfavorable physiological roles has inspired efforts to engineer l-lactate-specific genetically encoded fluorescent biosensors. Such biosensors could be powerful tools for studying the concentration dynamics of l-lactate in cells and tissues. The archetype of such biosensors is the Förster resonance energy transfer (FRET)-based biosensor known as Laconic.^19^ Laconic is composed of a cyan fluorescent protein (FP) and a yellow FP fused to the termini of the l-lactate-binding transcription factor LldR.^20^ It has been effectively used in a variety of applications including investigations of the Warburg effect^19,21^ and the astrocyte–neuron l-lactate shuttle (ANLS) hypothesis.^22,23^ Drawbacks of Laconic include its very small ratiometric response and its use of two colors of FP, limiting the opportunities for multiplexed imaging with other colors of biosensors.

Relative to FRET-based indicators, single FP-based indicators can typically be engineered to have much larger intensiometric fluorescence responses and are suitable for multiplexed imaging applications. In efforts aimed at realizing these advantages, a number of single FP-based l-lactate biosensors have been engineered in recent years.^24−31^ These single FP-based biosensors share a general design in which the l-lactate binding protein is genetically linked to an FP such that the binding protein is located close to the chromophore.^32^ Binding to l-lactate causes a conformational change that changes the chromophore environment and, consequently, the intensity of the fluorescence. Representative examples include the eLACCO2.1 and R-eLACCO2 biosensors for extracellular l-lactate, both based on the TTHA0766 l-lactate binding protein.^24,25,31^ These biosensors offer high performance in the extracellular milieu, but since they have a strict requirement for Ca^2+^ (10s of μM), they do not function in the cytosolic environment where the Ca^2+^ concentration is <1 μM. The existing LldR-based biosensors do not suffer from this Ca^2+^ dependence since LldR does not require Ca^2+^ for binding to an l-lactate molecule, as recently demonstrated with a red fluorescent biosensor.^31^ The green fluorescent LldR-based biosensors reported to date have relatively limited fluorescence responses toward l-lactate.^26,27^

Here we report a series of three new single GFP-based biosensors for l-lactate that overcome the limitations of previously reported biosensors and have affinities that span the physiological concentration range of l-lactate. These biosensors, designated iLACCO1, iLACCO1.1, and iLACCO1.2, are the final products of extensive directed evolution and structure-guided mutagenesis. As we demonstrate in this work, the iLACCO biosensors provide outstanding performance that greatly facilitates imaging of intracellular l-lactate dynamics in mammalian cells.

## Results

### Development of a Genetically Encoded l-Lactate Biosensor, iLACCO1

**Figure 1:**
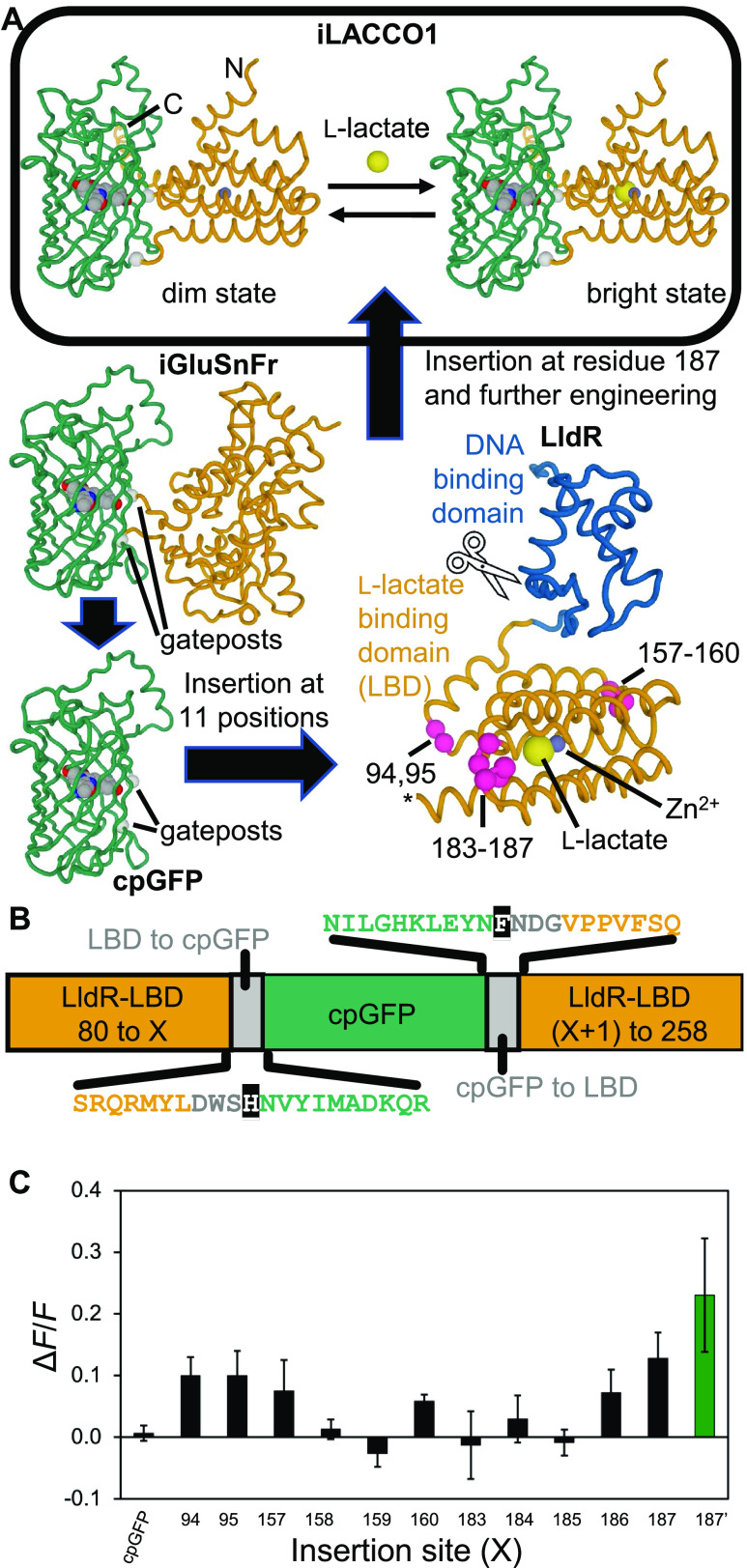
iLACCO design strategy. (A) Schematic representation of the overall strategy used to engineer iLACCO1. Structures shown are AlphaFold^34,35^ models of iLACCO1, iGluSnFr,^33^ cpGFP, and *E. coli* LldR. The Zn^2+^ (purple sphere) and l-lactate (yellow sphere) were positioned based on a superposition with the sialic acid-binding homologue NanR (PDB ID: 6ON4).^36^ Gate post residues demarcate the beginning and end of the cpGFP domain. Pink spheres represent insertion sites that were initially tested. To remove the N-terminal DNA-binding domain, the region of DNA encoding the first 79 residues of LldR was removed. (B) Schematic representation of the 11 insertion site variants initially tested. Linker regions are represented in gray. Gate posts are represented with white text on a black background. (C) Δ*F*/*F* of each prototype biosensor, where cpGFP is inserted at the site of LldR-LBD indicated on the horizontal axis. A variant with the insertion of cpGFP at site 187, which also had a point mutation in the second linker (NDG to NEG) (187′; green bar), gave the largest absolute value of Δ*F*/*F*, so this protein was designated iLACCO0.1. *n* = 3 technical replicates, mean ± s.d.

An initial prototype of the l-lactate biosensor was constructed by inserting circularly permuted green fluorescent protein (cpGFP), derived from iGluSnFR,^33^ into the l-lactate binding domain (LBD) of the *Escherichia coli* LldR transcriptional regulator protein^20^ at various solvent-accessible positions (Figure 1A). These insertion sites in LldR-LBD were chosen manually based on an AlphaFold model of the structure^34,35^ and were solvent-exposed sites that were considered likely to undergo l-lactate-dependent conformational changes. A total of 11 different insertion sites, located in 3 different solvent-accessible loops, were tested. Two linkers (each with three residues), DWS at the N-terminus (first linker) and NDG at the C-terminus (second linker) of cpGFP, were introduced to connect the cpGFP domain to the LldR-LBD (Figure 1B). These linkers, which are derived from eLACCO1,^24^ are connected to the two “gate post” residues of cpGFP (His145 and Phe148 as numbered in GFP; His110 and Phe352 as numbered in Figure S1). These gate post residues have been proposed to play an important role in cpGFP-based biosensors.^32^

In the process of cloning a variant with cpGFP inserted at position 187, a single colony (designated 187’) was found to be much brighter than others. This clone was later determined to have a point mutation in the second linker (NDG to NEG). This variant had the largest change in fluorescence intensity [Δ*F*/*F* = (*F*_max_ – *F*_min_)/*F*_min_] upon adding l-lactate and exhibited a direct response (fluorescence increase upon binding) to l-lactate of Δ*F*/*F* = 0.23 (Figure 1C). This variant was designated iLACCO0.1 and used as the template for further engineering.

To further develop iLACCO0.1 to obtain variants with larger absolute Δ*F*/*F* values, we first optimized the linker lengths (Figure S2A). Site-directed mutagenesis was used to obtain variants with partial or total deletions of both the first and second linkers (Figure S2B). The variant with all three amino acids of the first linker deleted and all residues of the second linker retained was determined to have the greatest response to l-lactate and was designated iLACCO0.2. This variant exhibited an inverse response (fluorescence decrease upon binding) with Δ*F*/*F* = −0.55 (Figure S2C).

**Figure 2:**
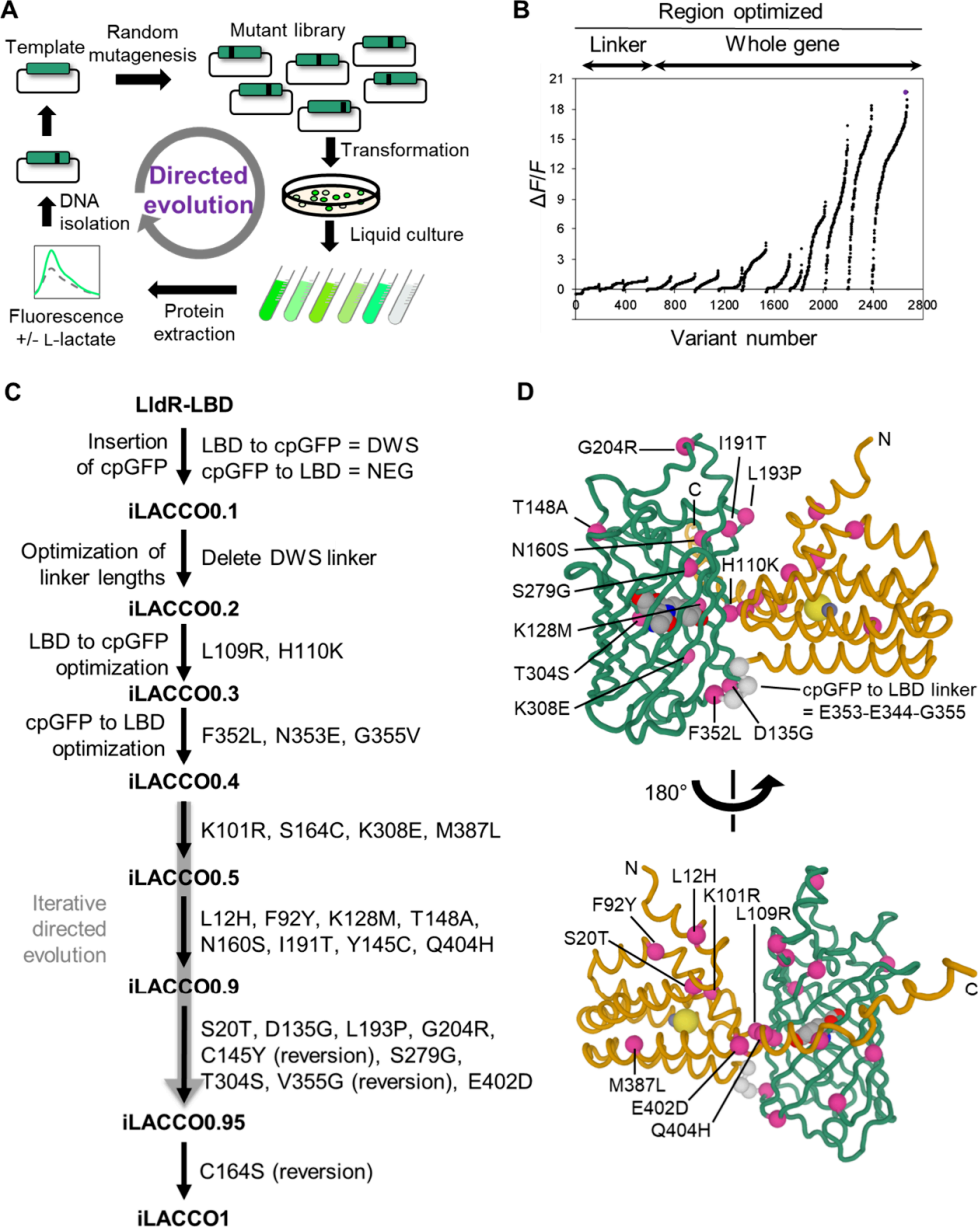
Directed evolution of iLACCO1. (A) Schematic of directed evolution workflow. Starting from the template of iLACCO0.4, the full-length gene was randomly mutated by error-prone PCR and the resulting library was used to transform *E. coli*. Bright colonies were picked and cultured, and Δ*F*/*F* upon addition of 10 mM l-lactate was determined using crude protein extracts. The genes encoding the variants with the highest Δ*F*/*F* were used as the template for the next round. (B) Δ*F*/*F* rank plot representing all proteins tested during the directed evolution. For each round, tested variants are ranked from lowest to highest Δ*F*/*F* value from left to right. (C) Lineage of iLACCO variants from LldR-LBD. (D) Modeled structure^34,35^ of iLACCO1 with the position of mutations indicated.

To further improve the fluorescence response, we next optimized the linker sequences and then performed directed evolution of the whole gene. Since the first linker had been deleted, three amino acids (Met107, Tyr108, and Leu109) of LldR-LBD, adjacent to the gate post residue His110, were considered to be the new N-terminal linker. We constructed and screened a series of libraries in which pairs of residues, in either the N-terminal or C-terminal linker, were sequentially randomized. Successive screening of these libraries led to the discovery of iLACCO0.4 with a direct response (Δ*F*/*F* = 1.3) to l-lactate. Further optimization was performed using directed evolution as shown schematically in Figure 2A. Eleven rounds of directed evolution led to the final iLACCO1 variant with Δ*F*/*F* = 20 under the screening conditions (i.e., crude protein extract in B-PER solution) (Figure 2B). Relative to iLACCO0.1, iLACCO1 contains 21 point mutations (Figures 2C,D and S1).

### *In Vitro* Characterization of iLACCO1

**Figure 3:**
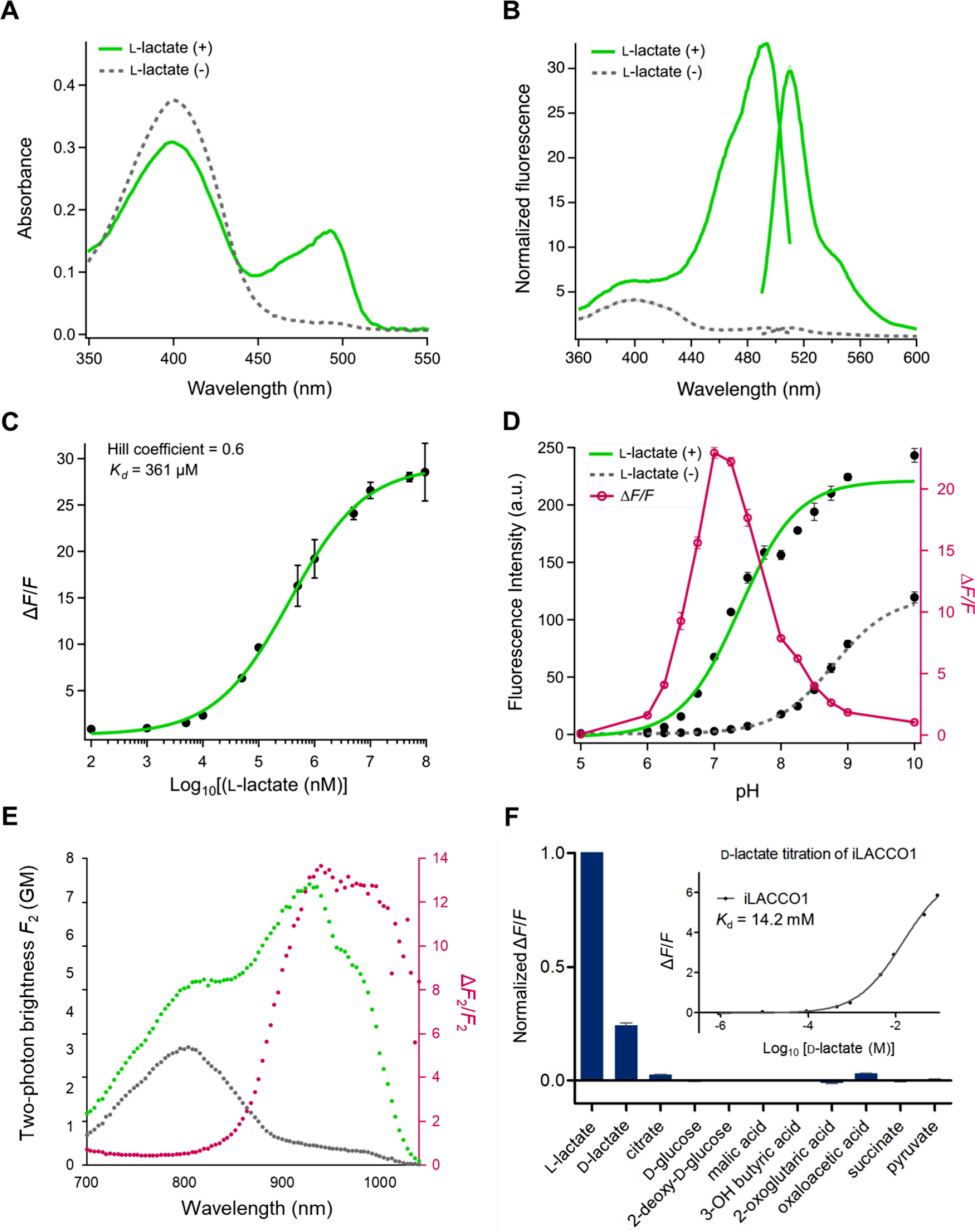
*In vitro* characterization of iLACCO1. (A) Absorbance spectra of iLACCO1 in the presence (10 mM) and absence of l-lactate. (B) Excitation (emission at 570 nm) and emission spectra (excitation at 450 nm) of iLACCO1 in the presence (95 mM) and the absence of l-lactate. (C) Dose–response curve of iLACCO1 for l-lactate. *n* = 3 technical replicates (mean ± s.d.). (D) pH titration curve of iLACCO1 in the presence (10 mM) and the absence of l-lactate. *n* = 3 technical replicates (mean ± s.d.). (E) Two-photon excitation spectra of iLACCO1 in the presence (10 mM) (represented in green dots) and absence of l-lactate (represented in gray dots) shown with the GM values label on the left *Y* axis. Δ*F*_2_/*F*_2_ is the ratio of the two-photon excitation spectra (represented in magenta dots) labeling the right *Y* axis. (F) Molecular specificity (9 mM each) of iLACCO1 and dose–response curve of iLACCO1 for d-lactate. *n* = 3 technical replicates (mean ± s.d.).

Characterization of key photophysical and biochemical properties of purified iLACCO1 revealed it to be a high-performance biosensor. In the presence of l-lactate, iLACCO1 exhibits absorbance peaks at 400 and 493 nm (Figure 3A), corresponding to the neutral (protonated) and the anionic (deprotonated) forms of the chromophore, respectively. Stopped flow analysis revealed that the kinetics of the l-lactate-dependent fluorescence response were complex, made up of at least two component steps: an immediate fluorescence response that occurs in the first 100 ms of mixing, followed by a longer increase that occurs over the course of minutes. At saturating concentrations of l-lactate, the immediate increase in fluorescence (<100 ms) of iLACCO1 accounts for about 75% of the total rise, iLACCO1.1 (see below) in <75 ms accounts for approximately 50% of the total rise, and iLACCO1.2 (see below) in <100 ms accounts for approximately 80% of the total rise. For all three variants, the fluorescence continues to increase slowly over the course of 10 min (Figure S3A,D,E). Steady-state absorption spectroscopy of iLACCO1, conducted over an extended time course, not only confirmed the kinetics of the second step but also revealed the third step, which is approximately 3 orders of magnitude slower than the second step (Figure S3B,C). The excitation spectrum of iLACCO1 in the presence of l-lactate has a maximum at 493 nm, consistent with the absorbance spectrum, and the emission maximum is 510 nm (Figure 3B). Purified iLACCO1 has a Δ*F*/*F* of 30 (Figure 3B) and an apparent dissociation constant (*K*_d_) of 361 μM (Figure 3C) for l-lactate at pH 7.2. iLACCO1 also exhibits p*K*_a_ values of 7.4 and 8.8 in the presence and absence of l-lactate, respectively (Figure 3D). The two-photon spectrum of iLACCO1 reveals that the excitation maximum of the l-lactate bound state is 928 nm (where the 2P absorption is dominated by the anionic form) with brightness of *F*_2_ = 7.3 GM (1 GM = 10^–50^ cm^4^ s; *F*_2_ ≃ σ_2,A_ × φ_A_ × ρ_A_, where the two-photon absorption cross section, σ_2,A_, the fluorescence quantum yield, φ_A_, and the relative fraction, ρ_A_, all correspond to the anionic form of the chromophore). The Δ*F*_2_/*F*_2_ value ranges from 13.0 to 14.7 in the 928–1000 nm wavelength range (Figure 3E).

*In vitro* testing revealed that iLACCO1 is highly specific for l-lactate and has a negligible fluorescence response to the structurally similar molecules and representative metabolites listed in Figure 3F. iLACCO1 does exhibit a substantial fluorescence response to its enantiomer, d-lactate, though with a 40× lower affinity (*K*_d_ = 14.2 mM; Figure 3F inset). Notably, the physiological concentration of d-lactate in plasma is tens of μM,^37^ which is hundreds of times lower than the apparent *K*_d_ of iLACCO1 for d-lactate. Note that the concentration of d-lactate was mistakenly stated to be in the nM range in an oft-cited 2005 review.^38^ Due to its lower affinity and the lower physiological concentration, d-lactate is unlikely to interfere with iLACCO-based measurements of l-lactate concentration.

### Development of iLACCO Variants with Different Affinities

**Figure 4:**
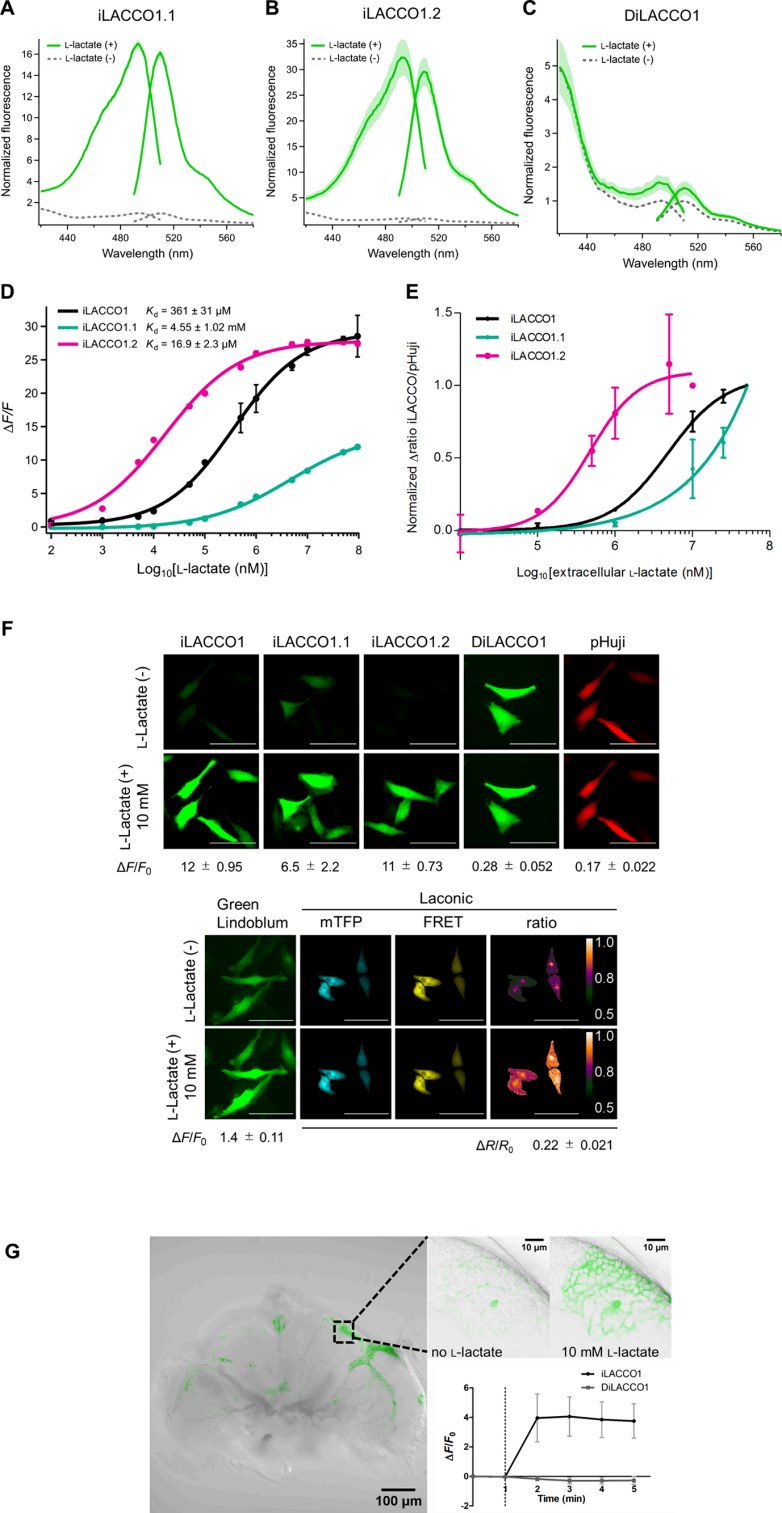
Characterization and demonstrations of iLACCO affinity series. (A–C) Excitation and emission spectra of iLACCO variants in the presence (95 mM) and the absence of l-lactate. *n* = 3 technical replicates (mean ± s.d.). (D) Dose–response curves of purified iLACCO1 variants upon treatment with l-lactate. *n* = 3 technical replicates (mean ± s.d.). (E) Dose–response curves of HeLa cells expressing iLACCO1 variants in response to treatments with extracellular l-lactate, as measured using flow cytometry. iLACCO1, 1.1, and 1.2 gave 50% of their maximal response at treatment concentrations of 4.8 mM, >10 mM, and 0.47 mM, respectively. *n* = 3 from independent experiments (mean ± s.d.), and around 1.0 × 10^5^ cells were analyzed for each independent experiment. (F) Fluorescent images of HeLa cells expressing iLACCO variants, pHuji,^40^ Green Lindoblum,^26^ and Laconic^19^ in the presence (10 mM) and absence of l-lactate. Δ*F*/*F*_0_ and Δ*R*/*R*_0_ are calculated from cells in the images shown. Scale bars represent 100 μm. *F*_0_ and *R*_0_ are determined as average fluorescence intensities of 15 data points before the addition of any reagent. (G) Glia cells of *Drosophila melanogaster* expressing iLACCO1. The image on the left is the whole brain (20× objective), and the images on the right are a close-up view before and after the addition of 10 mM l-lactate (63× objective). The grayscale image was inverted using ImageJ. The graph shows the Δ*F*/*F*_0_ of iLACCO1 (black) and DiLACCO1 (gray) during imaging. l-Lactate (10 mM) was added right after the snapshot at 1 min. *n* = 3, mean ± s. d.

The range of physiological concentration of intracellular l-lactate depends on cell types.^22^ In parallel with the process of directed evolution which ultimately produced iLACCO1, we also undertook the development of variants with different affinities for their wider applicability. Based on the X-ray crystal structure of LldR from *Corynebacterium glutamicum*,^20^ we constructed a homology model of LldR from *E. coli* using M4T Server ver. 3.0 (accessed on June 10, 2019).^39^ Based on the homology model, the l-lactate binding cavity of LldR is lined with hydrophobic residues that likely interact with l-lactate and charged residues that likely interact with a nonexchangeable zinc ion that coordinates with l-lactate.^20^ Focusing on the hydrophobic interactions, we designed a series of 10 conservative mutations (E39Q, D69E, M89Q, F93Y, L96I, V100T, V100I, L364I, V393T, and V393) that could potentially have an effect on the l-lactate binding affinity (Figure S4A). Each of these 10 mutations was individually introduced by site-directed mutagenesis into iLACCO0.5, and affinities for l-lactate were measured. Mutations which changed the affinity but did not abolish the fluorescence response (V100T, V100I, V393T, and V393I) were selected for further investigation (Figure S4B). Testing these mutations in the context of iLACCO0.9 enabled us to identify Val393Ile as the best mutation for decreased affinity (*K*_d_ = 4.55 mM) and Val100Ile as the best mutation for increased affinity (*K*_d_ = 16.9 μM) (Figure S4C). Finally, these mutations were introduced into iLACCO1 to produce iLACCO1.1 (iLACCO1 V393I; low affinity) and iLACCO1.2 (iLACCO1 V100I; high affinity) (Figure 4A,B).

As with most genetically encoded biosensors, the fluorescence of iLACCO1 and its variants is highly sensitive to pH changes within the physiological range. Control biosensors that do not respond to the target of interest but do exhibit a pH sensitivity similar to that of the biosensor (in one state or the other) are useful tools for helping researchers distinguish a true response from a pH-induced artifact. Having found that mutations of Val100 and Val393 can affect l-lactate binding, further mutations were introduced in these sites. The combination of Val100Ala and Val393Ala abolished the fluorescence response to l-lactate. This variant, designated “dead” iLACCO1 (DiLACCO1), showed a pH dependence that was similar to that of the lactate-free state of iLACCO variants and was used as the control biosensor (Figures 3D, 4C, and S5). The dose–response curves of the iLACCO variants toward l-lactate are shown in Figure 4D, and the two-photon spectra of iLACCO1.1 and iLACCO1.2 are shown in Figure S6. The photophysical and biochemical properties are summarized in Table S1.

### Characterization of iLACCO1 Variants in Mammalian Cells

iLACCO1 variants were characterized in HeLa cells using flow cytometry and fluorescence microscopy. We cloned each of the iLACCO genes into a mammalian expression vector with a CMV promoter. As a spectrally orthogonal control for possible pH changes, to the 3′ end of the iLACCO gene we appended the gene for the red fluorescent protein (RFP)-based pH biosensor pHuji,^40^ separated by a self-cleaving P2A sequence.^41^ To assess the functions of iLACCO1 variants based on large populations of cells, flow cytometry was conducted with cells that were expressing iLACCO variants and had been treated with various concentrations of l-lactate. Cells were treated with iodoacetic acid to stop intracellular l-lactate production, nigericin to clamp the pH, and rotenone to block mitochondrial metabolism. These conditions have previously been employed for the characterization of an l-lactate biosensor.^19^ Based on the dose–response curves of iLACCO-expressing cells, iLACCO1, 1.1, and 1.2 gave 50% of their maximal response at treatment concentrations of 4.8 mM, >10 mM, and 0.47 mM l-lactate, respectively (Figure 4E).

As another validation of the responses of the iLACCO variants in cells, we performed fluorescence microscopy of HeLa cells that were treated identically to the cells in the cytometry experiments. For the cells shown in Figure 4F, the observed Δ*F*/*F*_0_ values were 12 ± 0.95 for iLACCO1, 6.5 ± 2.2 for iLACCO1.1, 11 ± 0.73 for iLACCO1.2, 0.28 ± 0.052 for DiLACCO1, and 1.4 ± 0.11 for Green Lindoblum.^26^ For Laconic,^19^ Δ*R*/*R*_0_ was 0.22 ± 0.021, and for the pHuji pH indicator as a control, Δ*F*/*F*_0_ was 0.17 ± 0.022, which indicated that the pH change is negligible. Based on this data, we conclude that the iLACCO series of biosensors retains high performance in cells.

### *Ex Vivo* Imaging of l-Lactate in *Drosophila.*

To determine if iLACCO1 retained its performance in an *ex vivo* fly tissue, we created a transgenic UAS-iLACCO1 line and crossed with *repo-Gal4* to express iLACCO1 specifically in glial cells. Acutely isolated brains were incubated in modified HL3 media supplemented with 5 mM d-glucose, 1 mM l-lactate, and 0.5 mM pyruvate. After confirming the fluorescence expression of the biosensors in glia cells, the medium was exchanged for modified HL3 buffer containing 6 mM oxamate to induce so-called transacceleration,^42^ which is a process by which intracellular l-lactate is exported due to the import of oxamate. Time-lapse imaging was then carried out, and 10 mM l-lactate (final concentration) was added during imaging (Figure 4G). Upon the addition of 10 mM l-lactate, iLACCO1 exhibited Δ*F*/*F*_0_ = 4.1 within 1 min. This fluorescence intensity change is somewhat smaller than that with purified proteins, which may be due to competition with oxamate for binding to the biosensor. Under identical conditions, the control biosensor DiLACCO1 exhibited Δ*F*/*F*_0_ = −0.3. This experiment demonstrates that iLACCO1 can be functionally expressed in brain tissues of transgenic *Drosophila* and retains its high performance.

### Monitoring Intracellular l-Lactate with iLACCO Variants in Mammalian Cells

**Figure 5:**
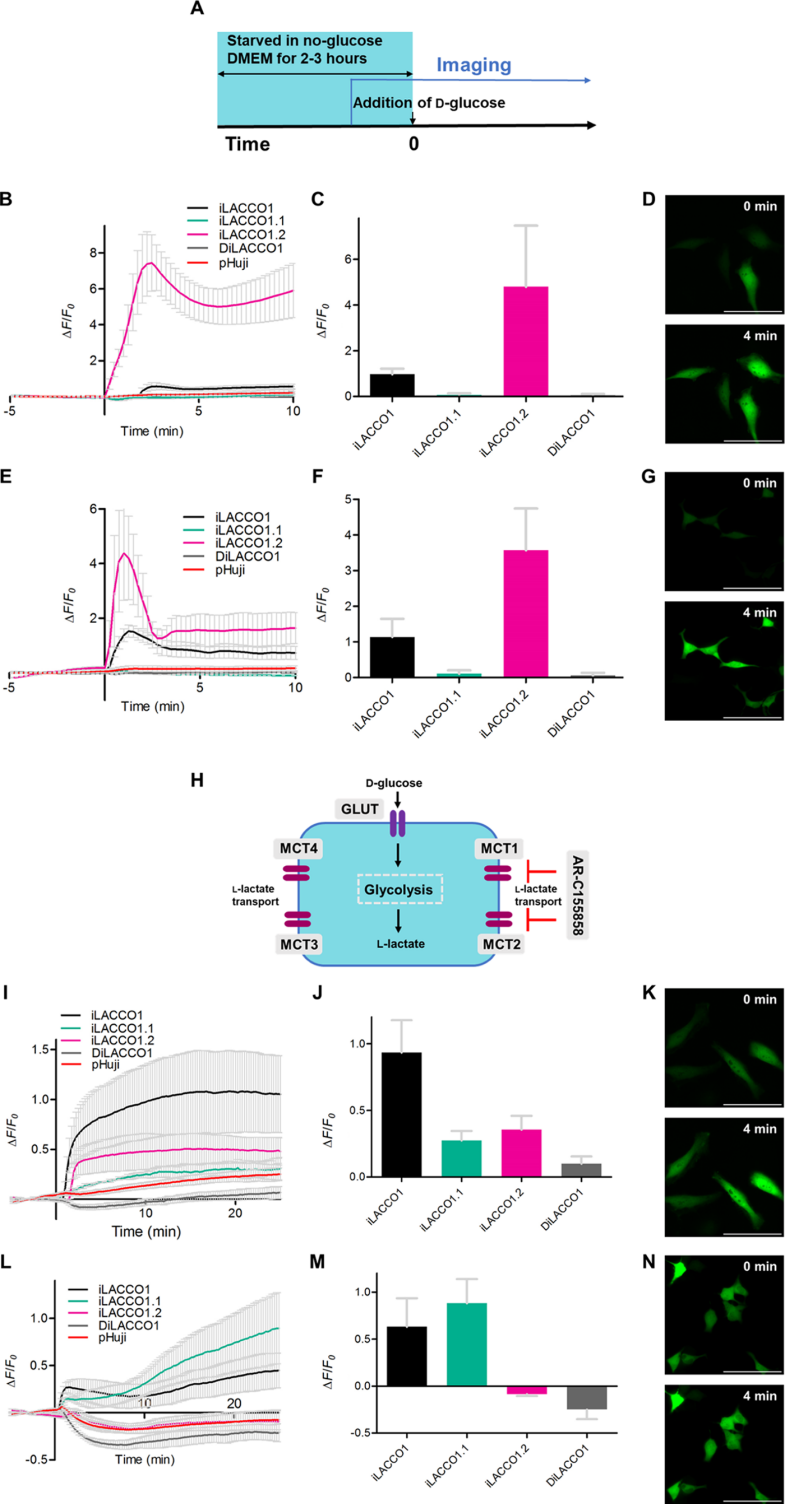
Imaging of iLACCO variants in starved and MCT-inhibited mammalian cells. (A) Schematic of imaging conditions of the starvation experiment. HeLa and HEK293 cells were starved in no-glucose medium for 2 to 3 h and were treated with a final concentration of 5 mM d-glucose at time = 0. Glucose-induced changes in the intracellular l-lactate concentration were observed with iLACCO1 variants expressed in HeLa (data shown in B–D) and HEK293 (data shown in E–G) cells. (B, E) Representative time courses show mean ± s.d. iLACCO1 (black, *n* = 6 and 3 cells for HeLa and HEK293, respectively), iLACCO1.1 (green, *n* = 5 and 2 cells for HeLa and HEK293, respectively), iLACCO1.2 (pink, *n* = 5 and 3 cells for HeLa and HEK293, respectively), and DiLACCO1 (gray, *n* = 5 and 4 cells for HeLa and HEK293, respectively) from a single independent experiment and pHuji (red, *n* = 21 and 12 cells for HeLa and HEK293, respectively). (C, F) Bar graphs show the mean ± s.d of maximum Δ*F*/*F*_0_ determined as the peak values within 5 min after the addition of d-glucose. iLACCO1 = (23, 6), iLACCO1,1 = (21, 6), iLACCO1.2 (20, 5), and DiLACCO1 = (10, 3), where (*x*, *y*) = (number of cells in total, number of independent experiments). (D, G) Representative images of HeLa cells expressing iLACCO1.2 (data shown in D) and HEK293 cells expressing iLACCO1.2 (data shown in G) before and after the treatment. Scale bars represent 100 μm. (H) Schematic of the MCT1,2 inhibitor experiment. The fluorescence intensity change was observed during the addition of AR-C155858 in HeLa (data shown in I–K) and HEK293 (data shown in L–N) cells. (I, L) Fluorescence response of iLACCO1 variants expressing HeLa and HEK293 cells upon treatment of MCT1,2 inhibitor AR-C155858. AR-C155858 (final 1 μM) was added at 0 min under a high glucose (25 mM) condition. Mean ± s.d. iLACCO1 (black, *n* = 4 and 4 cells for HeLa and HEK293, respectively), iLACCO1.1 (green, *n* = 2 and 5 cells for HeLa and HEK293, respectively), iLACCO1.2 (pink, *n* = 3 and 6 cells for HeLa and HEK293, respectively), and DiLACCO1 (gray, *n* = 4 and 4 cells for HeLa and HEK293, respectively) from a single independent experiment and pHuji (red, *n* = 13 and 20 cells for HeLa and HEK293, respectively). (J, M) Bar graphs show the mean ± s.d values of Δ*F*/*F*_0_ during time = 15 to 25 min for iLACCO1 = (22, 5), iLACCO1,1 = (16, 5), iLACCO1.2 (17, 4), and DiLACCO1 = (15, 5), where (*x*, *y*) = (number of cells in total, number of independent experiments). (K, M) Representative images of HeLa cells expressing iLACCO1 (data shown in K) and HEK293 cells expressing iLACCO1.1 (data shown in M) before and after the treatment. Scale bars represent 100 μm.

To investigate the utility of iLACCO variants for the monitoring of the l-lactate concentration in mammalian cells, we carried out imaging experiments using HeLa and HEK293 cells under several conditions. As shown in Figure 5A, cells were cultured in Dulbecco’s modified Eagle’s medium (DMEM) with high glucose (25 mM) and then 2 to 3 h before imaging the medium was switched to DMEM with no glucose. During imaging, glucose was added to the medium (*t* = 0) to a final concentration of 5 mM. We expected this treatment to cause an increase in intracellular l-lactate concentration and a corresponding increase in iLACCO fluorescence intensity, followed by a decrease as excess l-lactate is exported. In both cell lines, the high-affinity iLACCO1.2 gave the largest increase in fluorescence. iLACCO1 gave a substantially smaller change, and both iLACCO1.1 and DiLACCO1 gave negligible changes (Figure 5B–G). Assuming that the *K*_d_ values measured with purified protein (Figure 4D) are retained in the intracellular environment, these results are consistent with a baseline l-lactate concentration of 1 μM or less under starvation conditions, increasing to 10–100 μM upon treatment with 5 mM glucose.

To further investigate the utility of iLACCO variants for the monitoring of l-lactate concentration in mammalian cells, we examined the effect of an inhibitor of l-lactate flux through membrane transporters. AR-C155858 is a specific inhibitor^43^ of proton-coupled monocarboxylate transporters 1 and 2 (MCT1, MCT2), which transport l-lactate plus a proton across membranes (Figure 5H).^44^ We treated HeLa cells and HEK293 cells, transfected with iLACCO variants and cultured with high glucose, with this inhibitor and compared their fluorescence intensity changes. In HeLa cells, iLACCO1 (Δ*F*/*F*_0_ ≈ 0.89) showed a substantial increase in fluorescence intensity, while iLACCO1.1 (Δ*F*/*F*_0_ ≈ 0.25) and iLACCO1.2 (Δ*F*/*F*_0_ ≈ 0.28) had smaller increases by time = 15 to 25 min (Figure 5I–K). Assuming that the *K*_d_ values were measured with purified protein (Figure 4D), these results are consistent with an initial concentration of l-lactate in the range of hundreds of μM, increasing to 1 mM or greater upon treatment with AR-C155858. Both iLACCO1 and the higher-affinity iLACCO1.2 variant were observed to respond substantially faster than the lower-affinity iLACCO1.1 variant. In HEK293 cells, iLACCO1.1 exhibited the largest increase in fluorescence (Δ*F*/*F*_0_ ≈ 0.87), with iLACCO1 exhibiting a smaller but still substantial increase (Δ*F*/*F*_0_ ≈ 0.64). In contrast, iLACCO1.2 did not exhibit any substantial change (Figure 5L–N). These results are consistent with HEK293 cells having a higher baseline concentration (>1 mM) of intracellular l-lactate than HeLa cells under high glucose conditions. We also tested the expression of iLACCO1 in primary neurons and demonstrated that we could visualize an MCT inhibition-dependent increase in the intracellular l-lactate concentration (Figure S7).

### Intracellular l-Lactate Oscillations in Starved Mammalian Cells

**Figure 6:**
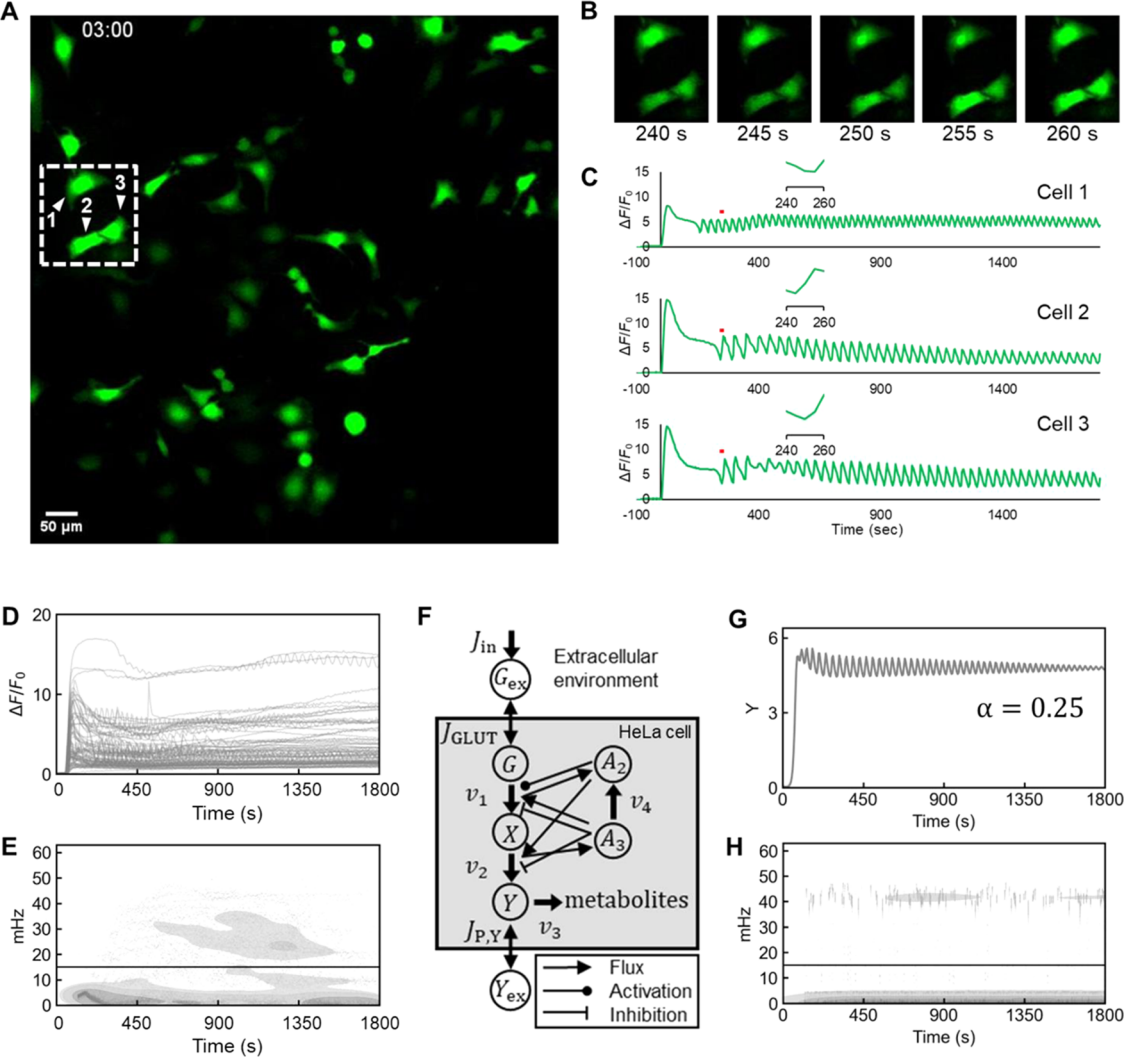
Characterization and modeling of l-lactate oscillations in HeLa cells. (A–D) Representative fluorescent images of HeLa cells expressing iLACCO1.2. (A, B) Snapshots from Movie S2 showing a whole field of view (A) and three cells at five time points (B). (C) Fluorescence response of iLACCO1.2 in selected cells versus time. Insets show the enlarged views of the time course at 240–260 s (indicated with red lines). (D) Experimental data of fluorescence versus time for 69 individual HeLa cells expressing iLACCO1.2, imaged in one experiment. HeLa cells were starved for around 4 h, and 5 mM d-glucose was used for treatment at *t* = 0. (E) 2D kernel density plot of instantaneous oscillatory frequencies. Oscillatory cells are defined as cells that oscillate at frequencies greater than 15 mHz (solid horizontal line). (F) Schematic representation of the model. The letters within circles indicate the metabolites of the model, the arrows indicate the fluxes, and the lines ending in circles or bars represent activation or inhibition, respectively. *G*_ex_ is extracellular glucose, *G* is intracellular glucose, *X* is intermediates after the PFK reaction, *Y* is l-lactate and other intermediates after the PK reaction, *Y*_ex_ is extracellular lactate, *A*_2_ is ADP, and *A*_3_ is ATP. Model and figure adapted from Amemiya et al.^47^ (G) One example of simulated *Y* (l-lactate and other intermediates after the PK reaction concentration) with α fixed at a value of 0.25 (Supporting Information). (H) Distribution of the simulated instantaneous frequency. Oscillatory cells are defined as cells that oscillate at frequencies greater than 15 mHz (solid horizontal line).

Glycolytic oscillations are well known to occur in starved cells treated with glucose.^45^ Following the addition of d-glucose to starved HEK293 and HeLa cells, we observed fluorescence oscillations in a small fraction of cells expressing either iLACCO1 or iLACCO1.2 (Figure 6A–C, Movies S1 and S2). We further investigated and found that longer starvation times (up to 4 h) increased the fraction of cells that were exhibiting oscillations. To obtain insight into this phenomenon, we acquired fluorescence imaging data for a large number of individual HeLa cells with iLACCO1.2 (Figure 6D, final concentration of 5 mM d-glucose was added at *t* = 0), performed a thorough statistical analysis of the data, and then attempted to model the results using a previously reported metabolic model.

To analyze the oscillation-like behavior of the fluorescence, we estimated the instantaneous frequencies and phases of the discrete Hilbert transforms of the temporal series (Figure S8A–D). Cells that exhibited oscillations at frequencies greater than 15 mHz (Figure 6E, the solid line), but below 50 mHz, were defined as the “oscillating cell” population. This frequency and occurrence (approximately half the cells; Figure S8E) is semiquantitatively consistent with oscillations in NADH concentration observed in previous studies.^46,47^ Synchronization analysis using the Kuramoto order parameter (*R*) revealed that the oscillations were asynchronous between cells (Figure S8F).^48^

In an effort to model the l-lactate oscillations, we turned to a previously reported mathematical model of glycolytic oscillations in HeLa cells (Figure 6F).^47^ This model includes rate constants for four chemical reactions (*v*_1_, *v*_2_, *v*_3_, *v*_4_) and three transport processes (*J*_in_, *J*_GLUT_, and *J*_P,Y_). Chemical reaction rates include the phosphofructokinase (PFK) reaction that represents upstream reactions in glycolysis (*v*_1_), the pyruvate kinase (PK) reaction that represents downstream reactions in glycolysis (*v*_2_), the overall rate of consumption reaction of the final products of glycolysis, including l-lactate (*v*_3_), and nonglycolytic ATP consumption (*v*_4_). Transport processes include the external input of glucose (*J*_in_), the transport of the extracellular glucose into the cell through glucose transporters (*J*_GLUT_), and the trans-membrane transport of triose including lactate (*J*_P,Y_). Other terms are defined in the Figure 6F legend.

We investigated how the previously reported range^47^ of variation in rate constants related to *J*_GLUT_, *v*_1_, *v*_2_, *v*_3_, and *v*_4_ affect the heterogeneity of the oscillations. Following the previously reported findings,^47^ the rate constants of the reactions (*k*_1_, *k*_2_, *k*_3_, and *k*_4_ for *v*_1_, *v*_2_, *v*_3_, and *v*_4_, respectively) were defined as functions of a single parameter α (Text S1). By simulating the instantaneous frequencies of l-lactate (*Y* in Figure 6F) concentration after glucose treatment with a range of α values (1000 simulations for each value with ±10% uniformly distributed random noise), we found that the ratio of oscillatory cells depends on the value of α (Figure S8G–J). When α was fixed at a value of 0.25, some cells exhibited oscillatory behavior, and Figure 6G depicts one such example. Time series analysis similar to Figure 6E suggested that some cells oscillated at frequencies greater than 15 mHz (Figure 6H, the solid line) and only approximately half of the cells exhibited oscillatory behavior, which is semiquantitatively consistent with the experimental data (Figures 6E and S8E). We also confirmed that the variations in the initial concentrations of intracellular glucose (*G*), intermediates after the PFK reaction (*X*), and l-lactate (*Y*) had little effect on the results (Figure S8K,L). Based on these experiments and simulations, we conclude that iLACCO1.2 enables the observation of glycolytic oscillations and that the heterogeneity of the oscillation-like behavior could be explained by a ±10% variation in the rate constants of the reactions.

## Discussion

We have developed a series of intensiometric, Ca^2+^-independent, genetically encoded, high-performance, single GFP-based biosensors for intracellular l-lactate, which we have designated as the iLACCO series. The initial prototype of this series was obtained by the insertion of cpGFP into a loop of the l-lactate binding domain (LBD) of the *E. coli* LldR transcriptional regulator protein. Starting from this prototype, we undertook extensive linker optimization and directed evolution to arrive at iLACCO1 with a Δ*F*/*F* of ∼30 upon binding to l-lactate. Notably, this is one of the highest fluorescence responses ever achieved for a single GFP-based biosensor for a ligand other than Ca^2+^ (ref (32)). Other recently reported GFP-based l-lactate biosensors have Δ*F*/*F* values of 0.88 (ref (27)), 1.9 (ref (29)), 4.2 (ref (26)), 6.0 (ref (24)), and 14 (ref (31)) (Table S2).

Site-directed mutagenesis of residues lining the modeled l-lactate binding pocket of the LldR domain resulted in variants with lower (iLACCO1.1) and higher (iLACCO1.2) affinity and high Δ*F*/*F* values of 15 and 28, respectively. Among these three variants, the detection of l-lactate concentrations in the range of ∼100 nM to ∼100 mM should be feasible in principle. The imaging applications with HeLa cells and HEK293 cells clearly demonstrate the utility of a series of iLACCO’s with different affinities that cover a wide dynamic range. Based simply on which affinity variant(s) gave the largest responses in a particular imaging experiment, we could make robust order-of-magnitude estimates of the l-lactate concentration changes, assuming the *K*_d_ values in cells are the same as for purified proteins.^50−53^

For single GFP-based biosensors, the fluorescence signal originating from the GFP is modulated by changes in the chromophore environment that occur as a result of conformational changes associated with ligand binding.^32^ Commonly, these changes in the chromophore environment cause a shift in the p*K*_a_ of the chromophore, leading to a change in the relative populations of the dim protonated state (phenol) and the bright deprotonated state (phenolate). The iLACCO series appears to employ just such a response mechanism. The absorbance spectrum of iLACCO1 in the absence of l-lactate reveals that the chromophore exists mostly in the protonated state (absorption peak at 400 nm) at neutral pH. Upon addition of l-lactate, the chromophore partially converts to the deprotonated form (absorption peak at 493 nm) (Figure 3A). This change is consistent with the observed shift in the p*K*_a_ of the iLACCO1 chromophore from 8.8 for the unbound state to 7.4 for the bound state (Figure 3D). This increase in the deprotonated state upon binding to l-lactate is the major contributing factor to the response of the biosensor. Notably, a large fraction of the protein remains in the protonated state even in the presence of l-lactate, suggesting that there is room for achieving much larger fluorescence responses, and higher brightness of the bound state, with further engineering.

Although an experimental atomic structure of iLACCO1 is not available, we can refer to an AlphaFold model^34,35^ of the protein and speculate on the molecular interactions that may be responsible for the l-lactate-dependent p*K*_a_ shift. Generally speaking, a shift from a higher to a lower p*K*_a_ could be attributable to a gain of new interactions that stabilize the deprotonated phenolate state (e.g., interaction with a positively charged group) or a loss of interactions that stabilize the protonated phenol state (e.g., interaction with a negatively charged or hydrophobic group). Intriguingly, the N-terminal gate post of iLACCO1 and its preceding residue are both positively charged (His110Lys and Leu109Arg, respectively). Furthermore, the C-terminal gate post and its following residue are both negatively charged (Asn353Glu and Glu354, respectively). Accordingly, we tentatively suggest that the l-lactate-dependent conformational change in the LBD is being propagated through the linkers to the cpGFP domain, leading to new interactions with the side chains of residues 109–110, and/or the loss of interactions with side chains of residues 353–354 (Figure S9). Further studies using X-ray crystallography and molecular dynamics simulations will likely be necessary to gain a better understanding of the fluorescence response mechanism.

A common disadvantage of most single GFP-based biosensors is pH sensitivity, and the iLACCO series is not an exception. The p*K*_a_ values for the l-lactate bound states of iLACCO1, iLACCO1.1, and iLACCO1.2 are 7.4, 7.7, and 6.8, respectively, values that are all in the physiological pH range. This pH sensitivity is potentially and particularly problematic for l-lactate biosensors because the MCTs transport l-lactate plus a proton, so l-lactate flux is necessarily associated with changes in pH. Specifically, l-lactate influx should be associated with a decrease in cytosolic pH, and l-lactate efflux should be associated with an increase in cytosolic pH. Fortunately, with respect to the iLACCO fluorescence intensity, the effect of decreasing pH (decreased fluorescence intensity) is opposite to the effect of increased l-lactate (increased fluorescence intensity). We recommend that control experiments with DiLACCO or the coexpression of a spectrally distinct pH biosensor, such as pHuji, should be routinely done when using the iLACCO series. Decreased sensitivity to pH changes could be an important feature to engineer into future iLACCO variants. As a precedent for such engineering, the l-lactate biosensor designated LiLac^28^ has been optimized for fluorescence lifetime imaging and minimal pH sensitivity.

When imaging starved HeLa cells that were treated with glucose, we observed oscillations in iLACCO fluorescence that were attributable to oscillations in the intracellular l-lactate concentration. The frequencies and the lack of synchronization for oscillations in the 15 to 50 mHz range were qualitatively consistent with previous studies.^46,47^ Metabolic modeling revealed that the existence of the oscillations and their distribution of frequencies were consistent with previously reported rates for relevant transport processes and enzymatic activities, assuming ±10% random variation. Unexpectedly, when starved cells were treated with a relatively low glucose concentration (500 μM), we found that they exhibited slow and synchronous oscillations with a period of approximately one oscillation per 450 s (Figure S8M–O). Previous studies have indicated that yeast cells may synchronize their glycolytic oscillations through the exchange of a metabolite acetaldehyde,^54,55^ whereas HeLa cells have been reported to exhibit weak intercellular synchronization,^46^ and the mechanism of intercellular synchronization in HeLa cells has not been reported. This synchronicity in HeLa cells may suggest a role for an extracellular signaling molecule, possibly l-lactate itself. Further investigations, possibly using multicolor imaging of multiple metabolite biosensors (e.g., l-lactate and pyruvate), targeted to the mitochondria or the cytosol, will be required to obtain mechanistic insight into this process and explore its possible physiological relevance.

In conclusion, we report a series of high-performance intracellular l-lactate biosensors that can be used to visualize intracellular l-lactate dynamics with large fluorescence responses and over a wide concentration range. We expect that the iLACCO series should be highly amenable to a broad range of further applications, including *in vivo* and *ex vivo* imaging to investigate physiological l-lactate concentration dynamics in animal models.

## Materials and Methods

### General Methods and Materials

A synthetic human codon-optimized gene encoding the *E. coli* LldR transcriptional regulator protein was purchased from Integrated DNA Technologies. Phusion high-fidelity DNA polymerase (Thermo Fisher Scientific) was used for routine polymerase chain reaction (PCR) amplification, and Taq DNA polymerase (New England Biolabs) was used for error-prone PCR. A QuickChange mutagenesis kit (Agilent Technologies) was used for site-directed mutagenesis. Restriction endonucleases and rapid DNA ligation kits (Thermo Fisher Scientific) were used for plasmid construction. Products of PCR and restriction digests were purified using agarose gel electrophoresis and the GeneJET gel extraction kit (Thermo Fisher Scientific). DNA sequences were analyzed by DNA sequence service of Fasmac Co. Ltd. The fluorescence spectra and intensity were recorded on Spark plate reader (Tecan) or a CLARIOstar Plus microplate reader (BMG LABTECH).

### Structural Modeling of LldR and iLACCO1

The modeling structure of LldR and iLACCO1 was generated by AlphaFold2 (refs (34) and (35)) using an API hosted at the Södinglab in which the MMseqs2 server^56^ was used for multiple sequence alignment (accessed on October 13, 2021). The amino acid sequence of the *E. coli* LldR transcriptional regulator protein was submitted to ColabFold to generate the modeling structure of LldR.^35^ The amino acid sequence of iLACCO0.2 was submitted to ColabFold to generate the template for the iLACCO1 structure. This specific sequence was chosen because it does not include newly introduced mutations which make the sequence identity closer to what can be found in the training data set of AlphaFold2.

### Engineering of iLACCO1 Variants

The gene encoding cpGFP with N- and C-terminal linkers (DWS and NDG, respectively) was amplified, followed by insertion into each site of LldR-LBD in a pBAD vector (Life Technologies) by Gibson assembly (New England Biolabs). The DNA binding domain of LldR was removed beforehand. Variants were expressed in *E. coli* strain DH10B (Thermo Fisher Scientific) in LB media supplemented with 100 μg mL^–1^ ampicillin and 0.02% l-arabinose. Proteins were extracted with the B-PER bacterial protein extraction reagent (Thermo Fisher Scientific) for the assay of fluorescence brightness and the l-lactate-dependent response at screening. During evolution processes, the MnCl_2_ concentration was controlled to obtain one to two mutations in the whole gene per round. Primary screening was done on the agar plates, where approximately 2 × 10^3^ colonies were visually inspected each round. A total of 192 bright colonies were then picked up for protein extraction and fluorescence measurement. After 3 rounds of linker optimization, 11 rounds of directed evolution in the whole gene sequence followed by the introduction of the C164S mutation ultimately led to iLACCO1. Mutations for tuning affinity were introduced by site-directed mutagenesis using the QuikChange mutagenesis kit.

### Protein Purification

iLACCO variants in pBAD expression vectors containing a N-terminal poly His (6×) tag were expressed in *E. coli* BL21(DE3). A single colony was used to inoculate a 10 mL culture of 100 μg mL^–1^ ampicillin LB and grown overnight at 37 °C by shaking at 180 rpm. Saturated culture (1 mL) was then used to inoculate 1 L of LB supplemented with 100 μg mL^–1^ ampicillin and grown at 37 °C until an OD_600_ value of 0.6 was reached. The cell culture was then induced by adding 0.1% (w/v) l-arabinose and grown overnight at 18 °C. The next day, cells were harvested by centrifugation at 4 °C and 4,000 rpm for 1 h. Each gram of cell pellet was resuspended in 6 mL of lysis buffer (30 mM MOPS, 100 mM KCl, 10% (v/v) glycerol, 1 mM TCEP, 1 mM PMSF, 5 mM benzamidine, 10 mM imidazole, pH 7.2). Cells were placed on ice and lysed by sonication (30 s on/off for 2.5 min, 2.5 min off, and 30 s on/off for 2.5 min) and then centrifuged at 12,000 rpm and 4 °C for 1 h. Lysates were filtered with a 0.45 μm filter and loaded onto a lysis buffer pre-equilibrated Ni-NTA column. The column was then washed with 10 column volumes (cv) of lysis buffer. Protein was eluted with 3 cv of elution buffer (30 mM MOPS, 100 mM KCl, 2% (v/v) glycerol, 1 mM TCEP, 0.2 mM PMSF, 0.2 mM benzamidine, 500 mM imidazole, pH 7.2) and buffer exchanged into l-lactate (−) buffer (30 mM MOPS, 100 mM KCl, pH 7.2) using a centrifugal spin column (10K MWCO, Thermo Fisher Scientific). Prior to analysis, all proteins were then further purified by size exclusion chromatography using a Superdex 75 10/300 GL increase column (GE Healthcare).

### *In Vitro* Characterization

Absorption spectra were collected on SPECTROstar Nano microplate reader (BMG LABTECH) using a 10 mm quartz cuvette (Hellma Analytics). To measure the interaction between iLACCO1 and l-lactate through steady-state absorption spectroscopy, absorption at 493 nm was detected through a 10 mm quartz cuvette using a diode array UV–vis spectrophotometer (Ocean Optic Inc., USB4000). Absorption data was collected every 100 ms for 10 s (integration time: 30 ms, scans to average: 3, boxcar width: 25). The time courses were fitted with *f*(*t*) = *c* + *A* (1 – exp(−*k*_obs_*t*)). To perform rapid kinetic measurement for the interaction between iLACCOs and l-lactate, equal volume of 0.2 μM sensor protein were mixed with varying concentrations of l-lactate in an Applied Photophysics SX20 stopped flow fluorimeter with 490 nm LED excitation and a 510 nm long-pass filter at room temperature (22 °C). Each mixing was repeated five times (except for 10 min experiments, which were collected only once) and averaged. The first 3 ms of data was not analyzed to remove mixing artifacts and accounts for the dead time of the instrument. Data was plotted, and time courses were attempted to be fit (Kaleidagraph version 5.01, Synergy Software) to a single rising exponential (*y* intercept + total rise(1 – exp(−*k*_obs_*t*))). When the time course did not fit well to a single rising exponential, it was fit to the sum of two rising exponentials (*y* intercept + first rise(1 – exp(−*k*_obs1_*t*)) + second rise(1 – exp(−*k*_obs2_*t*))). All fluorescence spectra were collected on a CLARIOstar Plus Microplate reader (BMG LABTECH) using a Greiner 96-well flat-bottom microplate. For absorption and fluorescence excitation/emission spectra, l-lactate (−) buffer and l-lactate (+) buffer (30 mM MOPS, 100 mM KCl, 100 mM l-lactate, pH 7.2) were used. To measure *K*_d_, a series of buffers with l-lactate concentration ranging from 0 to 100 mM were prepared by diluting l-lactate (+) buffer using l-lactate (−) buffer. The sensitivity of the sensors as a function of l-lactate concentration was then fitted to the Hill equation (*f*(*x*) = base + (max-base)/(1 + (*K*_d_/*x*)^*n*^)) to determine the Hill coefficient (*n*) and apparent *K*_d_. The sensitivity of the sensors is reported as Δ*F*/*F*, which is calculated with (*F*_*x*_ – *F*_(−)_)/*F*_(−)_, where *F*_*x*_ is the fluorescence intensity at 510 nm of sample *x* and *F*_(−)_ is the fluorescence intensity at 510 nm of the same concentration of protein in l-lactate (−) buffer. For pH titration, pH buffer (30 mM MOPS, 30 mM trisodium citrate, 30 mM sodium borate, 100 mM KCl, and either no l-lactate or 10 mM l-lactate, with pH ranging from 5 to 10) was used to dilute protein solutions. Fluorescence intensities as a function of pH were then fitted to a sigmoidal function (*f*(pH) = base + (max-base)/(1 + 10^p*K*_a_–pH^)) to determine the p*K*_a_. All measurements were conducted at room temperature.

### Two-Photon Absorption Measurements

Two-photon excitation spectra and two-photon absorption cross sections were measured using standard methods and protocols.^57^ Briefly, tunable femtosecond laser InSight DeepSee (Spectra-Physics, Santa Clara, CA) was used to excite the fluorescence of the sample in a PC1 spectrofluorometer (ISS, Champaign, IL). To measure the two-photon excitation spectral shapes, we used in the emission channel a combination of short-pass filters 633SP and 770SP for iLACCO1 and iLACCO1.2 and an additional 535/50 filter for iLACCO1.1. Organic dyes LDS 798 in 1:2 CHCl_3_:CDCl_3_ and Coumarin 540A in DMSO were used as spectral standards. The quadratic power dependence of fluorescence intensity in the proteins and standards was checked at several wavelengths across the spectrum.

The two-photon cross section (σ_2_) of the anionic form of the chromophore was measured as described previously.^58^ Fluorescein in water at pH 12 was used as a reference standard with excitation at 900 nm.^57^ For one-photon excitation, we used a 488 nm line of an argon ion laser (Melles Griot), and a combination of filters 770SP and 520LP was in the fluorescence channel. Extinction coefficients were determined by alkaline denaturation as previously described.^59^ The two-photon absorption spectra were normalized to the measured σ_2_ values. To normalize to the total two-photon brightness (*F*_2_), the spectra were multiplied by the quantum yield and the relative fraction of the anionic form of the chromophore. The data is presented this way because iLACCO1, iLACCO1.1, and iLACCO1.2 all exist as mixtures of the neutral and anionic forms of the chromophore at neutral pH. The method has been previously described in detail.^59^

### Construction of Mammalian Expression Vectors

The gene of an iLACCO variant was amplified with sequence coding P2A self-cleaving peptide by PCR and cut with XhoI and *Eco*RI. The gene encoding pHuji^40^ was amplified by PCR, followed by digestion with *Eco*RI and *Hin*dIII. Finally, these products were ligated into the pcDNA3 vector (Thermo Fisher Scientific) with T4 ligase (Thermo Fisher Scientific). The genes of Laconic (Addgene plasmid no. 44238) and Green Lindoblum (synthetic DNA purchased from Integrated DNA Technologies) were ligated into a pcDNA3 vector without pHuji.

### Imaging of iLACCO Variants in Mammalian Cells

HeLa [Japanese Cancer Research Resources Bank (JCRB) and American Type Culture Collection (ATCC)] and HEK293 (JCRB) cells were maintained in Dulbecco’s modified Eagle’s medium (DMEM high glucose; Nacalai Tesque) supplemented with 10% fetal bovine serum (FBS; Sigma-Aldrich) and 100 μg mL^–1^ penicillin and streptomycin (Nacalai Tesque). Cells were transiently transfected with the plasmids with polyethylenimine (Polysciences) in Opti-MEM (Gibco) and imaged within 48–72 h after transfection. An IX83 wide-field fluorescence microscope (Olympus) equipped with a pE-300 LED light source (CoolLED) and a 40× objective lens (numerical aperture (NA) = 1.3; oil), an ImagEM X2 EM-CCD camera (Hamamatsu), and Cellsens software (Olympus) was used for the imaging. The filter sets in the imaging had the following specifications. iLACCO1 variants and Green Lindoblum: excitation 470/20 nm, dichroic mirror 490 nm dclp, and emission 518/45 nm; pHuji: excitation 545/20 nm, dichroic mirror 565 nm dclp, and emission 598/55 nm; Laconic (mTFP1): excitation 438/24 nm, dichroic mirror 458 nm dclp, and emission 483/32 nm; and Laconic (FRET): excitation 438/24 nm, dichroic mirror 458 nm dclp, and emission 542/27 nm. Fluorescent images were analyzed with ImageJ software (https://imagej.net/software/fiji/, National Institutes of Health; accessed on September 1, 2023).

In starvation experiments, HeLa and HEK293 cells were incubated in no-glucose DMEM (Nacalai Tesque) for 2–4 h before imaging. After exchanging medium in no-glucose imaging buffer (184.45 mg L^–1^ CaCl_2_·H_2_O, 97.6 mg L^–1^ MgSO_4_, 400.00 mg L^–1^ KCl, 60.00 mg L^–1^ KH_2_PO_4_, 8000.00 mg L^–1^ NaCl, 350.00 mg L^–1^ NaHCO_3_, 47.88 mg L^–1^ Na_2_HPO_4_), a final concentration of 5 mM or 500 μM d-glucose was added at time = 0 min. Methods describing the analysis and modeling of l-lactate oscillations are provided as Supporting Information.

For imaging in treatment with MCT inhibitor AR-C155858 (Tocris), Hank’s balanced salt solution (HBSS; Nacalai Tesque) supplemented with additional d-glucose (final concentration of 25 mM) and 10 mM HEPES (Nacalai Tesque) was used as an imaging buffer.

Neuronal imaging was performed as previously reported.^24^ All methods for animal care and use were approved by the institutional review committees of the School of Science, The University of Tokyo. Briefly, rat cortical/hippocampal primary cultures from P0 pups (pooled tissues from males and females) from a single timed-pregnant Sprague–Dawley rat (Charles River Laboratories, purchased from Japan SLC, Inc.) were plated in glass-bottomed 24-well plates with 0.5 × 10^6^ cells for three wells. Cultures were nucleofected at the time of plating with Nucleofector 4D (Lonza) and imaged 14 days later. The neuron culture was kept in NbActive4 (BrainBits) media and exchanged into imaging buffer (145 mM NaCl, 2.5 mM KCl, 10 mM d-glucose, 10 mM HEPES, 2 mM CaCl_2_, 1 mM MgCl_2_, pH 7.4) prior to imaging.

### Flow Cytometry of HeLa Cells Expressing iLACCO Variants

Within 48–72 h after the transfection, cells were collected after incubation with 500 μM iodoacetic acid (the same amount of Milli-Q or none was added to the control samples) and washed with phosphate-buffered saline (PBS). The cells were suspended in HBSS supplemented with 10 mM HEPES and respective reagents (10 μM nigericin and 2 μM rotenone in stimulated samples) and were passed through a cell strainer with 35 μm mesh (Falcon). Flow cytometry analysis was carried out using SH800 (Sony). The data were analyzed with FlowJo software (BD).

### Transgenic Line Generation in *Drosophila melanogaster.*

The coding sequence of the sensor was amplified from pcDNA3.1 and inserted into the *Eco*RI and *XbaI* sites of the pUAST-attB vector using the In-Fusion Snap Assembly Master Mix (TAKARA). The transgenic lines were generated via Φ integrase-mediated recombination into the fly genome at landing site *attp2* (Rainbow Transgenic Flies Inc.).

### Imaging of *Ex Vivo Drosophila* Adult Brains

Imaging of *ex vivo Drosophila* adult brains (3–5 day old females) was performed at room temperature with an LSM800 confocal microscope (Zeiss). Adult brains were quickly dissected in Schneider’s Drosophila Medium (Gibco, 21720024) and then put on a polylysine-coated glass-bottomed dish (Matsunami Glass Ind. Ltd., D11131H) filled with modified HL3 buffer without CaCl_2_ or trehalose (70 mM NaCl, 5 mM KCl, 20 mM MgCl_2_, 10 mM NaHCO_3_, 115 mM sucrose, 5 mM HEPES; pH 7.2) supplemented with 5 mM d-glucose, 1 mM l-lactate, and 0.5 mM pyruvate. The dish was placed on the microscope stage, and the medium in the dish was replaced with a modified HL3 buffer containing 6 mM oxamate. Whole brain images were then captured with a 20× Plan-Apochromat objective (NA 0.8) in single plane mode (scan speed: 8; 1024 pixels × 1024 pixels; 8 bits per pixel; averaging number: 16; pinhole 1–1.5 AU). Time series images were taken every 60 s for 5 min with a 63× Plan-Apochromat oil DIC objective (NA 1.4) in *z*-stack mode (scan speed: 8; 1024 pixels × 1024 pixels; 8 bits per pixel; averaging number: 4; pinhole 1–1.5 AU; *z*-stack: 5 slices).

## Data Availability

The data and plasmids encoding iLACCO variants that support the findings of this study are available from the corresponding authors on reasonable request.

## Supporting Information Available

Supplementary Figures S1 to S9, legends for supplementary movies 1 and 2, supplementary tables S1 and S2, supplementary text describing the analysis and modeling of l-lactate oscillations, and supplementary references (PDF)
Starved HeLa cells expressing iLACCO1 were imaged (10× objective) for 385 frames (5 s/frame). HeLa cells were starved without FBS and d-glucose for 4 hours and final concentration of 5 mM d-glucose was added between frames 25 and 26 (*t* = 2 mins). The cell-free background intensity was subtracted from all frames using ImageJ. Imaging conditions are the same as for other starvation experiments described in the Materials and Methods section (AVI)
Starved HeLa cells expressing iLACCO1.2 were imaged (10× objective) for 385 frames (5 s/frame). HeLa cells were starved without FBS and d-glucose for 4 hours and final concentration of 5 mM d-glucose was added between frames 25 and 26 (*t* = 2 mins). The cell-free background intensity was subtracted from all frames using ImageJ. Imaging conditions are the same as for other starvation experiments described in the Materials and Methods section (AVI)

## Supplementary Material

oc3c01250_si_001.pdf

oc3c01250_si_002.avi

oc3c01250_si_003.avi
