# Supplementary material for: High-Performance Genetically Encoded Green Fluorescent Biosensors for Intracellular l-Lactate
Source: ACS Cent Sci. 2024 Jan 31;10(2):402–16. doi: 10.1021/acscentsci.3c01250 (PMC10906044; doi:10.1021/acscentsci.3c01250)
Supplement: Supplementary file 1 — oc3c01250_si_001.pdf [file oc3c01250_si_001.pdf]

SUPPORTING INFORMATION FOR

High performance genetically-encoded green fluorescent biosensors for intracellular L-lactate

Saaya Hario[1†], Giang N. T. Le[1,2†], Hikaru Sugimoto[3], Kei Takahashi-Yamashiro[4,5], Suguru Nishinami[6], Hirofumi Toda[6], Selene Li[1], Jonathan S. Marvin[7], Shinya Kuroda[8], Mikhail Drobizhev[9], Takuya Terai[1], Yusuke Nasu[1,10*], and Robert E. Campbell[1,5,11*]

[1]Department of Chemistry, School of Science, The University of Tokyo, Bunkyo-ku, Tokyo 113-0033, Japan.

[2]Department of Chemistry, University of Toronto, Toronto, Ontario M5S 3H6, Canada.

[3]Department of Biochemistry and Molecular Biology, Graduate School of Medicine, The University of Tokyo, Bunkyo-ku, Tokyo 113-0033, Japan.

[4]Department of Molecular Pathology, Graduate School of Medicine, The University of Tokyo, Bunkyo-ku, Tokyo 113-0033, Japan.

[5]Department of Chemistry, Faculty of Science, University of Alberta, Edmonton, Alberta T6G 2G2, Canada.

[6]International Institute for Integrative Sleep Medicine, University of Tsukuba, Tsukuba, Ibaraki 305-8575, Japan.

[7]Howard Hughes Medical Institute, Janelia Research Campus, Ashburn, VA 20147, USA.

[8]Department of Biological Sciences, School of Science, University of Tokyo, Bunkyo-ku, Tokyo 113-0033, Japan.

[9]Department of Microbiology and Cell Biology, Montana State University, Bozeman, MT 59717, USA.

[10]PRESTO, Japan Science and Technology Agency, Chiyoda-ku, Tokyo 102-0075, Japan.

[11]CERVO Brain Research Center and Department of Biochemistry, Microbiology, and Bioinformatics, Université Laval, Québec, Québec G1V 0A6, Canada.

[†]These authors contributed equally and each one reserves the right to list their name first in their respective CVs.

*Corresponding authors. Email: nasu@chem.s.u-tokyo.ac.jp (Y.N.) and campbell@chem.s.u-tokyo.ac.jp (R.E.C.)

TABLE OF CONTENTS

SUPPLEMENTARY FIGURES S3

Figure S1. Sequence alignment of iLACCO1 ....S3

Figure S2. Optimization of linker lengths S4

Figure S3. Stop-flow analysis and steady-state absorption spectroscopy of iLACCO variants *in vitro*. S5

Figure S4. Engineering of affinity variants of iLACCO. S6

Figure S5. pH titration curve of iLACCO variants. S7

Figure S6. Two-photon excitation spectra of iLACCO1.1 and iLACCO1.2. ....S8

Figure S7. iLACCO1 expressed in a neuron treated with a MCT-inhibitor S9

Figure S8. Analysis and modeling of L-lactate oscillations. S10

Figure S9. Proposed response mechanism and chromophore interactions of iLACCO1. ....S12

SUPPLEMENTARY MOVIES LEGEND S13

SUPPLEMENTARY TABLES S14

Table S1. Photophysical and biochemical properties of the iLACCO series S14

Table S2. Summary of properties of reported FP-based L-lactate biosensors. S15

SUPPLEMENTARY TEXT S16

Analysis and modeling of L-lactate oscillations in HeLa cells. S16

SUPPLEMENTARY REFERENCES S19

SUPPLEMENTARY FIGURES

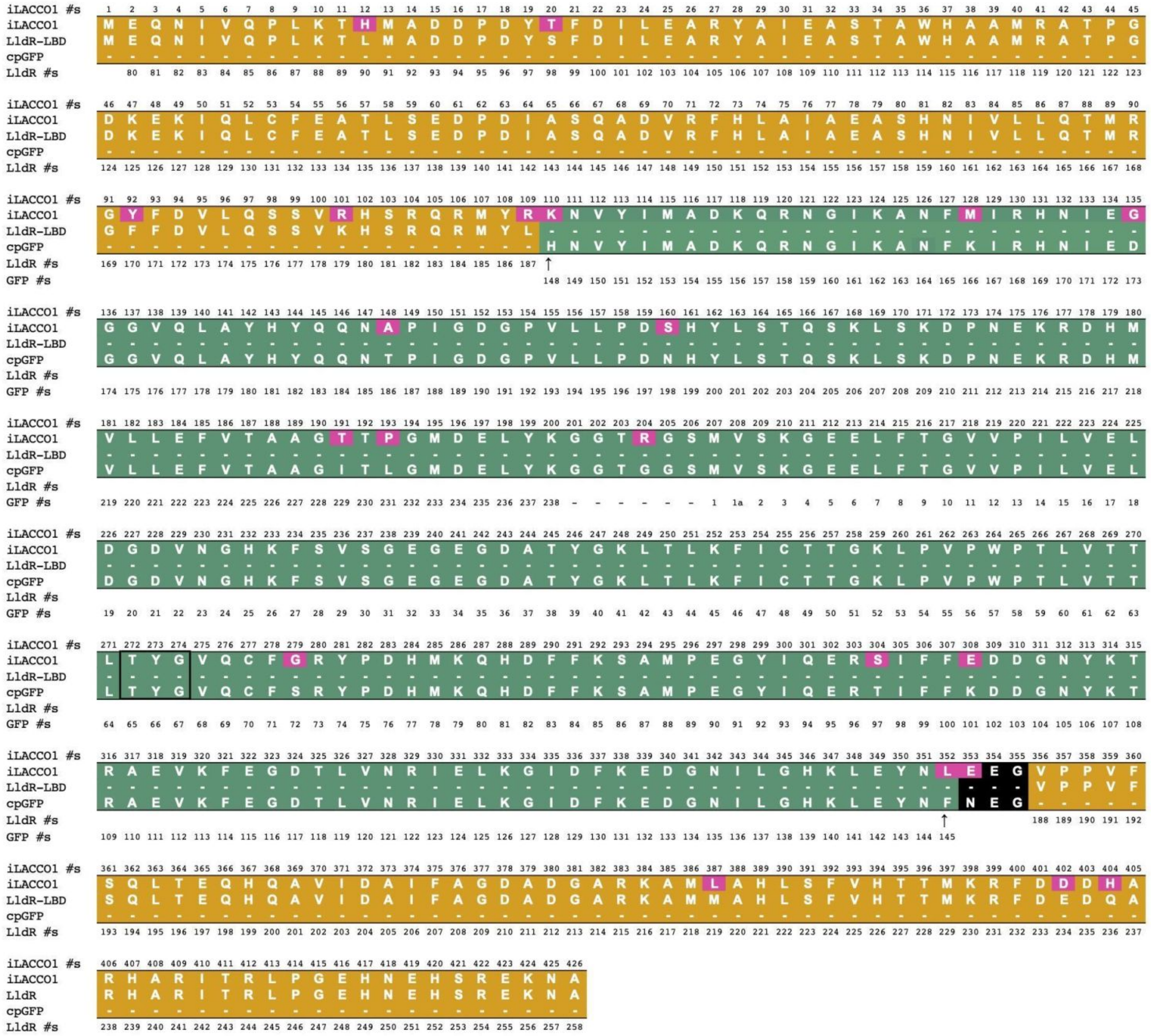

Figure S1. Sequence alignment of iLACCO1. The LldR-LBD domain is indicated by an orange background and the cpGFP domain is indicated by a green background. Mutations in iLACCO1, relative to LldR-LBD and cpGFP, are indicated by a magenta background. The linker at the C-terminal end of cpGFP is indicated by a black background. The gate post residues at the ends of cpGFP (residues 145 and 148), are indicated by upward pointing arrows. The linker joining the original C- and N-termini of GFP (residues 238 and 1, respectively) is not numbered. The chromophore-forming residues are enclosed in black box.

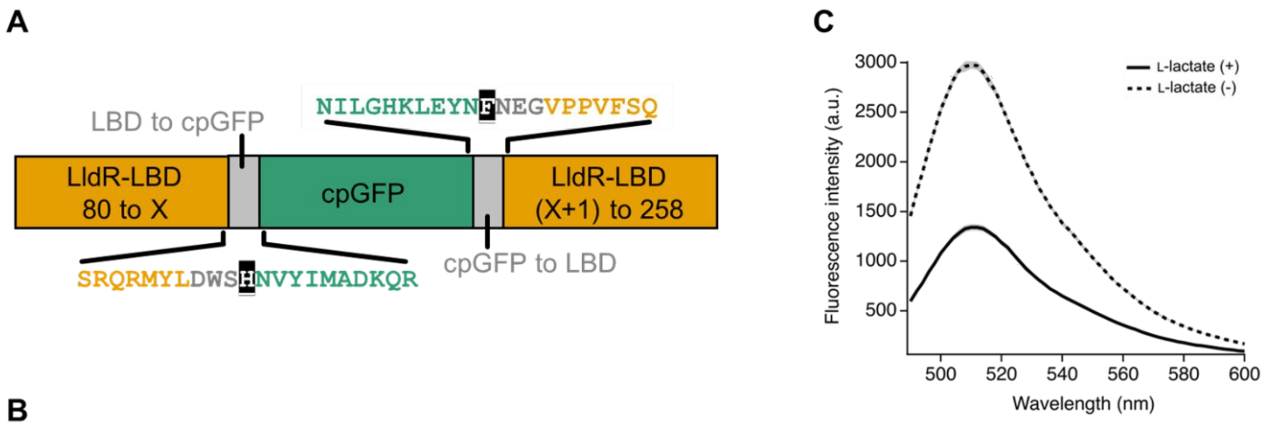

B

N-terminal linker \ C-terminal linker	NEG	-EG	NE-
DWS	0.14	0.02	ND
-WS	0.20	0.11	0.17
D-S	0.02	0.03	0.09
DW-	0.09	0.00	ND
--S	0.01	0.12	0.08
D--	0.01	0.09	0.08
--	0.55	0.06	0.02

$0<|\Delta F/F|\leq 0.1$

$0.1<|\Delta F/F|\leq 0.2$

$0.2<|\Delta F/F|$

Not Determined

Figure S2. Optimization of linker lengths. (A) Schematic representation of the primary structure of iLACCO0.1. iLACCO0.1 has DWS and NEG as N- and C-terminal linker, respectively. **(B)** Δ*F*/*F* profile of each linker length variant. The variant with no N-terminal linker and original C-terminal linker residues (NEG) showed the largest absolute value of Δ*F*/*F* and the protein was designated iLACCO0.2. **(C)** Emission spectra of iLACCO0.2 in the presence (10 mM, represented in solid line) and absence of L-lactate (represented in dashed line). *n* = 3 technical replicates (mean ± s.d.).

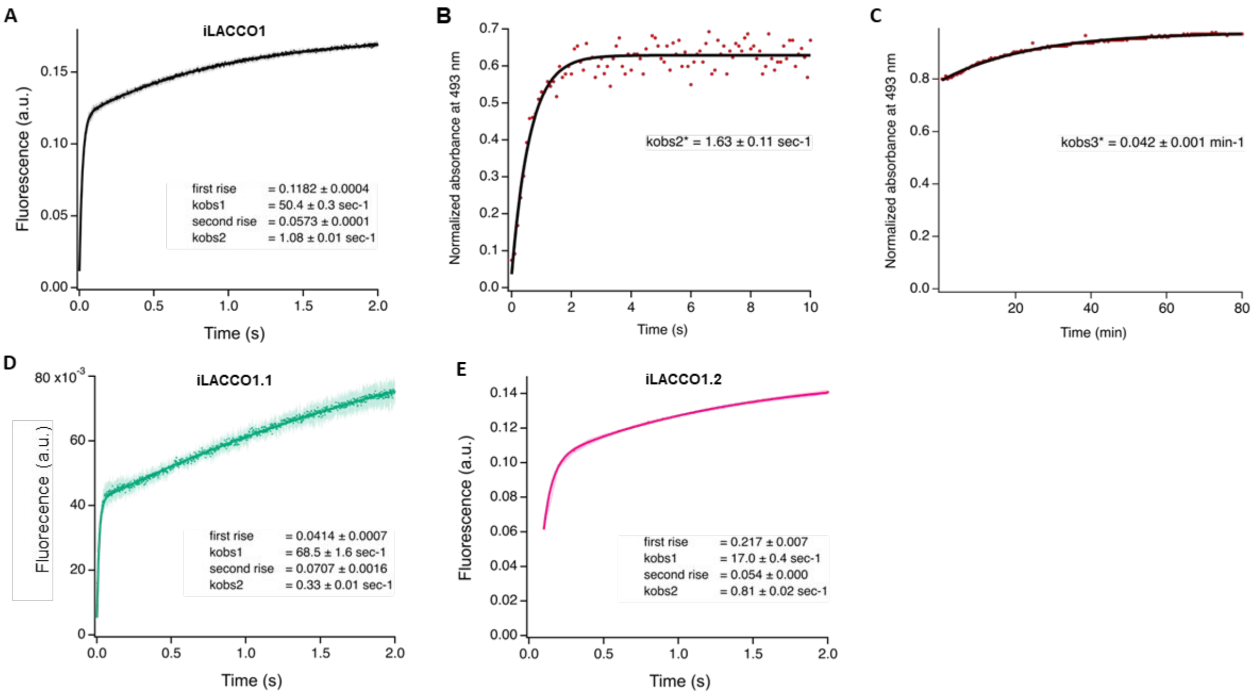

Figure S3. Stop-flow analysis and steady-state absorption spectroscopy of iLACCO variants *in vitro*. (A) Stop-flow fluorescence data of iLACCO1 (0.2 μM) upon mixing with 200 mM L-lactate. *n* = 5 technical replicates (mean ± s.d.). **(B-C)** Normalized absorbance at 493 nm of purified iLACCO1 protein upon adding 10 mM L-lactate as a function of time. **(D)** Stop-flow fluorescence data of iLACCO1.1 (0.2 μM) upon mixing with 200 mM L-lactate. *n* = 5 technical replicates (mean ± s.d.). **(E)** Stop-flow fluorescence data of iLACCO1.2 (0.2 μM) upon adding 2.5 mM L-lactate. *n* = 5 technical replicates (mean ± s.d.).

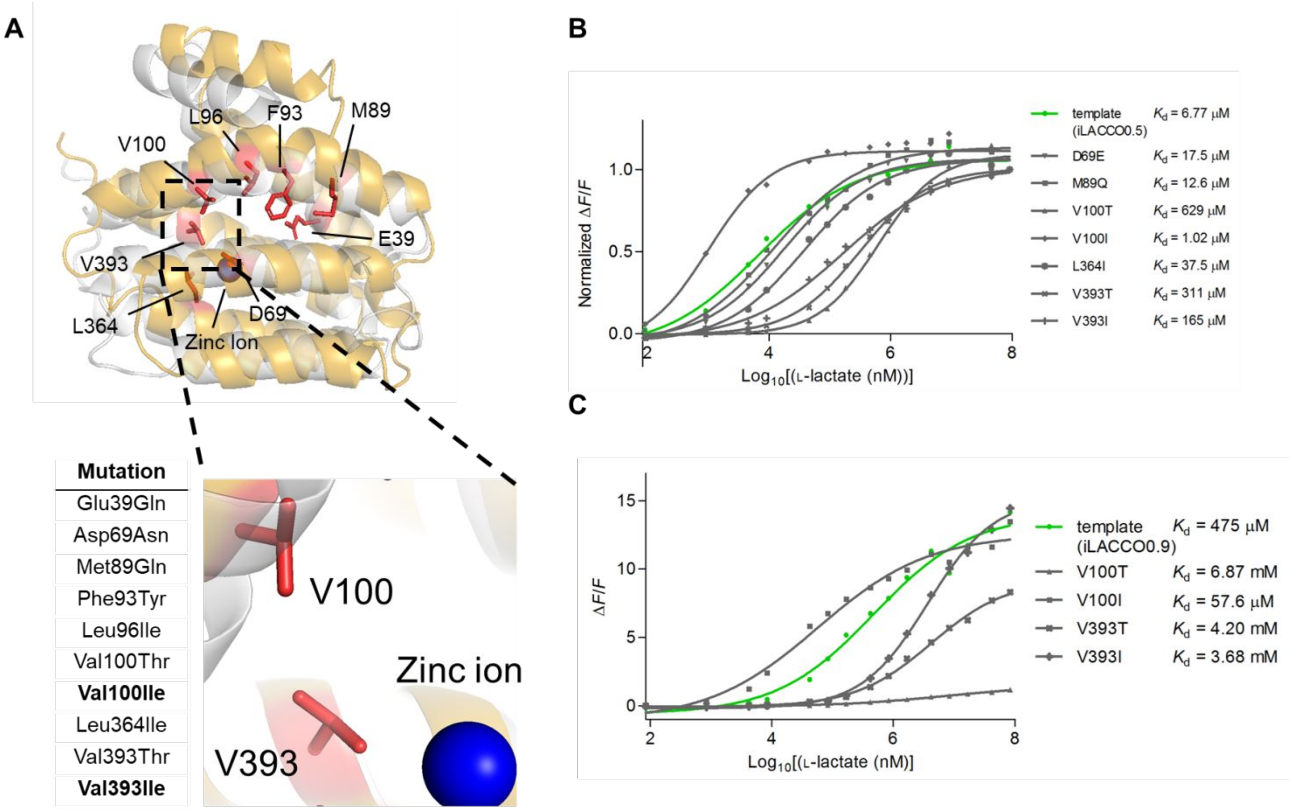

Figure S4. Engineering of affinity variants of iLACCO. (A) A homology model of LldR from *E. coli* (based on LldR from *Corynebacterium glutamicum*; PDB ID: 2DI3)[1] calculated on M4T Server ver. 3.0 (accessed on June 10, 2019)[2] (yellow), and superpositioned with the crystal structure of LldR from *C. glutamicum* (gray). Amino acid residues in the binding pocket that were mutated for possible affinity tuning are shown in red. The blue sphere indicates a Zn^{2+}. **(B)** Dose-response curves and calculated K_d values for mutants of iLACCO0.5 (n = 1, the same trend was confirmed in independent experiments). Three mutations (V100T, V393T, and V393I) that decreased the affinity and one mutation (V100I) that increased the affinity were selected for testing on later variants in directed evolution. **(C)** Dose-response curves and calculated K_d and $\Delta F/F$ values for mutated variants of iLACCO0.9 (n = 1, the same trend was confirmed in independent experiments). V100I was applied for tuning affinity higher and V393I was applied for tuning affinity lower. These mutations were later introduced to the final variant iLACCO1.

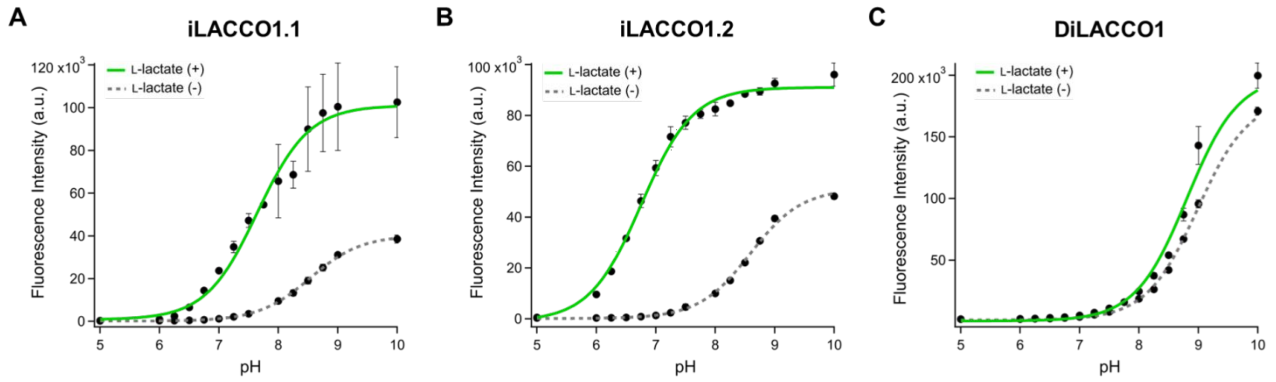

Figure S5. pH titration curve of iLACCO variants. pH titration curve of **(A)** iLACCO 1.1, **(B)** iLACCO1.2, and **(C)** DiLACCO1 in the presence (10 mM) and absence of L-lactate. *n* = 3 technical replicates (mean ± s.d.).

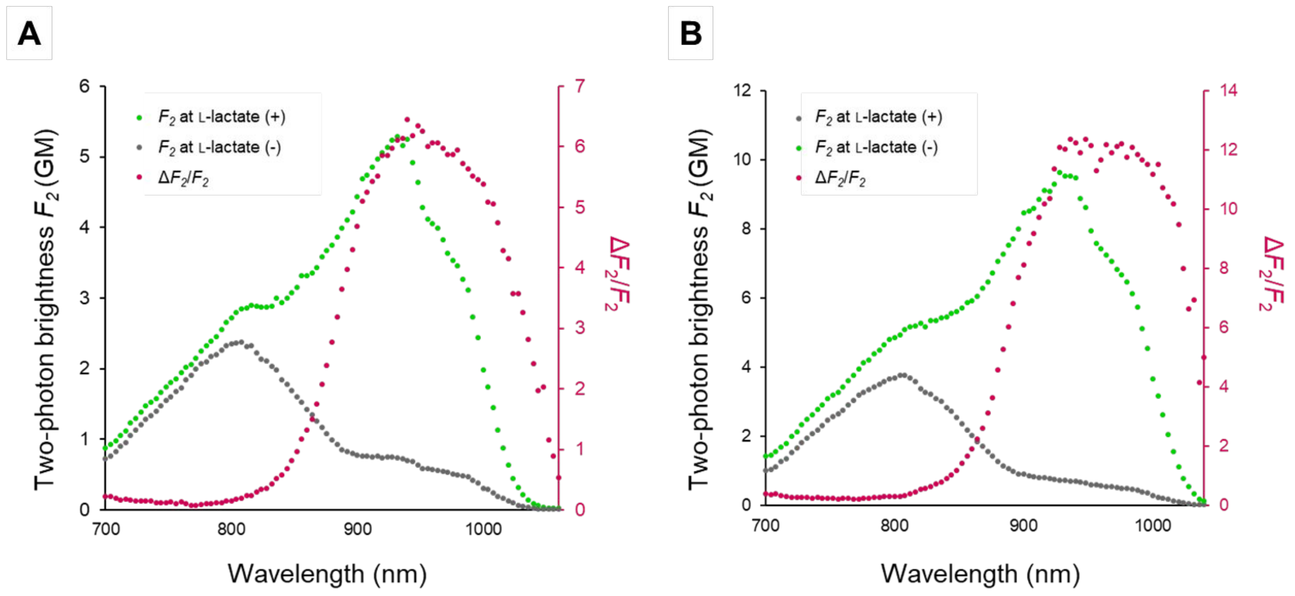

Figure S6. Two-photon excitation spectra of iLACCO1.1 and iLACCO1.2. Two-photon excitation spectra of **(A)** iLACCO1.1 and **(B)** iLACCO1.2 in the presence (10 mM) and absence of L-lactate.

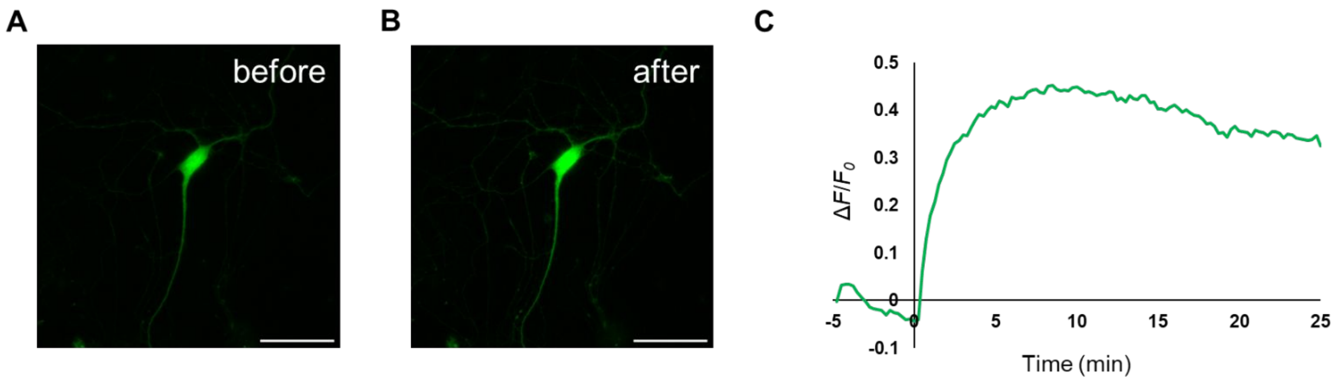

Figure S7. iLACCO1 expressed in a neuron treated with a MCT-inhibitor. (A-B) Representative images of hsyn-iLACCO1 expressed in a neuron **(A)** before and **(B)** after treatment with AR-C15585. **(C)** Fluorescence response upon treatment with AR-C15585 (final concentration 1 μM) added at *t* = 0 min under high glucose (25 mM) conditions. Scale bars represents 50 μm.

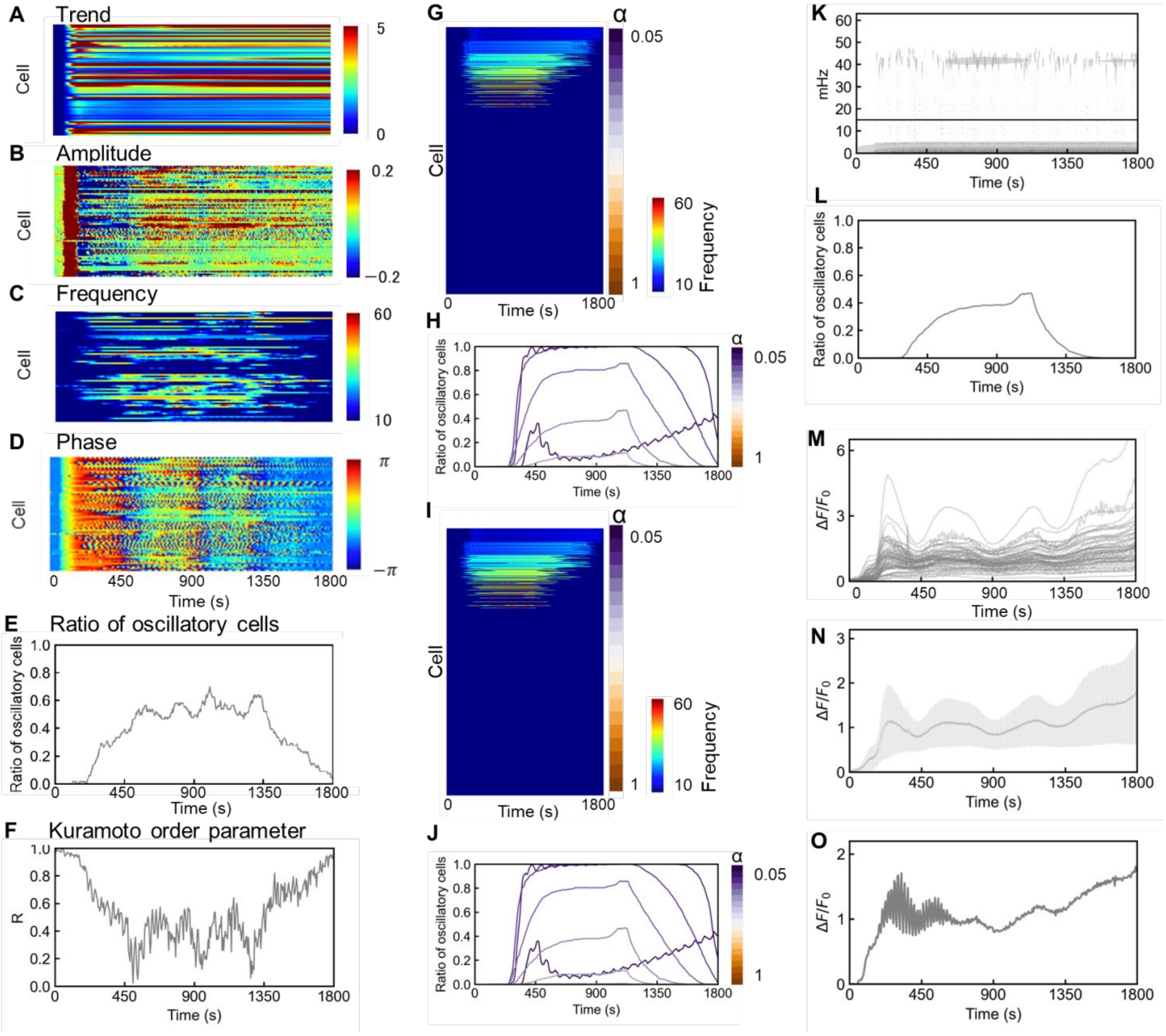

Figure S8. Analysis and modeling of L-lactate oscillations. Trend **(A)**, amplitude **(B)**, instantaneous frequency **(C)**, and instantaneous phase **(D)** of each cell represented in **Figure 6A**. For **A-D**, baseline trends were calculated as the walking average of the fluorescence data using a 100 s time window. Instantaneous phase was computed using the Hilbert transform. **(E)** Ratio of oscillatory cells (defined as cells that oscillate frequencies greater than 15 mHz) to the total cells. **(F)** Kuramoto order parameter (R) for the cell population. R is a normalized metric ranging from 0 to 1, with a value of 1 denoting complete synchronization of the population's oscillations. The R values were relatively low during most cells oscillating (see the interval from 450 s to 1350 s), indicating that the oscillations were asynchronous. **(G)** Heatmap of the instantaneous frequency of simulated lactate oscillations. The columns and the rows represent time after glucose administration and the cell, respectively. Each of the 1000 lines represents the characteristics of the frequency when α is increased from 0.05 to 1 in increments of 0.05. In each line, rate constants related to J_{GLUT}, v_1, v_2, v_3, and v_4 were uniformly randomized from 90% to 110%. **(H)** Simulated ratio of oscillatory cells. The colors of the lines are based on the values of α. **(I)** Heatmap of the instantaneous frequency of simulated lactate oscillations.

The columns and the rows represent time after glucose administration and the cell, respectively. Each of the 1000 lines represents the characteristics of the frequency when α is increased from 0.05 to 1 in increments of 0.05. In each line, rate constants related to J_{GLUT}, v_1, v_2, v_3, and v_4 were uniformly randomized from 90% to 110%. Moreover, the initial concentrations of *G*, *X*, and *Y* were generated from a Gaussian distribution with mean 0.3 and standard deviation 0.053. **(J)** Simulated ratio of oscillatory cells. The colors of the lines are based on the values of α. **(K)** Distribution of the simulated instantaneous frequency. "Oscillatory cells" are defined as cells that oscillate at the frequencies greater than 15 mHz (the solid line). **(I)** Simulated ratio of oscillatory cells. **(M)** Experimental data of iLACCO1.2 fluorescence ($\Delta F/F_0$) versus time for starved HeLa cells treated with 500 μM D-glucose (t = 0). **(N)** Average and standard deviation of iLACCO1.2 fluorescence ($\Delta F/F_0$) in **M**. **(O)** A representative example of a starved HeLa cell exhibiting both fast and slow lactate oscillations after the treatment with 500 μM D-glucose.

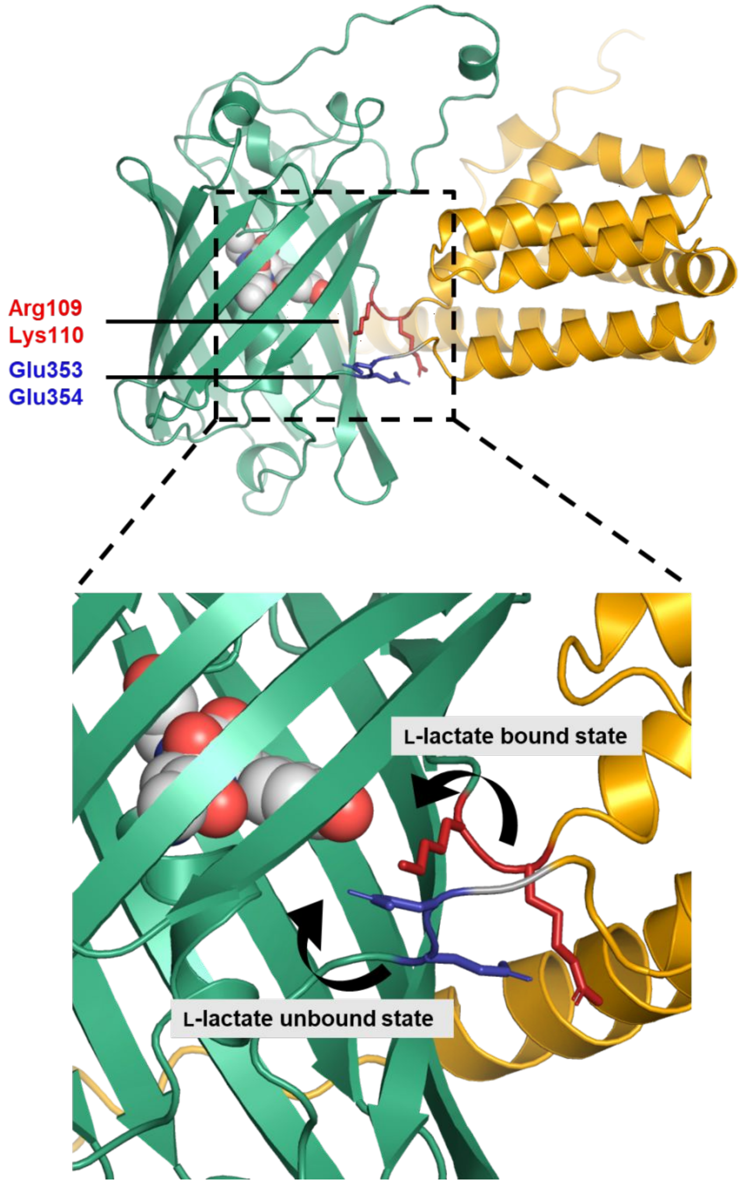

Figure S9. Proposed response mechanism and chromophore interactions of iLACCO1. Shown is an AlphaFold[3] model of iLACCO1 with the chromophore-forming tripeptide represented as spheres, the N-terminal gate post and its preceding residue (His110Lys and Leu109Arg, respectively) represented as red sticks, and the C-terminal gate post and its following residue (Asn353Glu and Glu354, respectively) represented as blue sticks. As described in the main text, we tentatively suggest that in the L-lactate-bound state, the positively charged residues shown in red are interacting with the chromophore and stabilizing in the anionic phenolate state. Conversely, in the L-lactate unbound state, the negatively charged residues shown in blue may be interacting with the chromophore and stabilizing it in the neutral phenol state.

SUPPLEMENTARY MOVIES LEGEND

Starved HeLa cells expressing iLACCO1 (**Movie S1**) and iLACCO1.2 (**Movie S2**) were imaged (10× objective) for 385 frames (5 sec/frame). HeLa cells were starved without FBS and D-glucose for 4 hours and final concentration of 5 mM D-glucose was added between frame 25 and 26 (t = 2 mins). Cell-free background intensity was subtracted from all frames using ImageJ. Imaging conditions are the same as for other starvation experiments described in the **Materials and Methods** section.

SUPPLEMENTARY TABLES

Table S1. Photophysical and biochemical properties of the iLACCO series.

	Key Mutations	$\Delta F/F$	K_d (mM)	+/- Lac	Excitation peak (nm)	Emission peak (nm)	pK_a	Anionic QY
**iLACCO1**	NA	29.7 ± 0.5	0.36 ± 0.03	+	493	510	7.38 ± 0.06	0.84 ± 0.01
				-	492	511	8.78 ± 0.03	0.72 ± 0.03
**iLACCO1.1**	V393I	15.2 ± 0.4	4.6 ± 1.0	+	493	510	7.65 ± 0.06	0.57 ± 0.07
				-	493	510	8.53 ± 0.01	0.57 ± 0.07
**iLACCO1.2**	V100I	28.4 ± 2.7	0.017 ± 0.002	+	493	509	6.76 ± 0.03	0.86 ± 0.01
				-	493	510	8.59 ± 0.02	0.61 ± 0.03
**DiLACCO1**	V100A V393A	0.4 ± 0.2	NA	+	493	510	8.81 ± 0.05	ND
				-	493	510	8.99 ± 0.02	ND

QY, quantum yield.
NA, not applicable.
ND, not determined.
n = 3 technical replicates (mean ± s.d.).

Table S2. Summary of properties of reported FP-based L-lactate biosensors.

Biosensor name	FP(s)	L-lactate binding protein (notes)	Response type (intra- or extracellular)	Maximum Response (conditions)	K_d (mM)	Ref.
***Aequorea* Green Fluorescent Protein (GFP)-based**						
iLACCO1	GFP	LldR (no DNA-binding domain)	Intensiometric (intracellular)	$\Delta F/F_0$ = 30 (purified protein)	0.36	This work
iLACCO1.1	GFP	LldR (no DNA-binding domain)	Intensiometric (intracellular)	$\Delta F/F_0$ = 15 (purified protein)	4.6	This work
iLACCO1.2	GFP	LldR (no DNA-binding domain)	Intensiometric (intracellular)	$\Delta F/F_0$ = 28 (purified protein)	0.017	This work
Green Lindoblum	GFP	LldR (no DNA-binding domain)	Intensiometric (intracellular)	$\Delta F/F_0$ = 4.2 (cell lysate)	0.030	4
C-GEM-IL 3.0	CFP	LldR (no DNA-binding domain)	Intensiometric (intracellular)	$\Delta F/F_0$ = 0.88 (purified protein)	0.66	5
CanlonicSF	GFP	TTHA0766 (Ca^{2+}-dependent)	Intensiometric (intracellular)	$\Delta F/F_0$ = 1.9 (cell lysate)	0.30	6
FiLa	YFP	LldR (no DNA-binding domain)	Excitation ratiometric (intracellular)	$\Delta R/R_0$ = 15 (purified protein)	0.13	7
LARS1.8	GFP	HCAR1 (not specific for lactate)	Intensiometric (extracellular)	$\Delta F/F_0$ = 0.3 (HEK293 cells)	1.5	8
eLACCO1	GFP	TTHA0766 (Ca^{2+}-dependent)	Intensiometric (extracellular)	$\Delta F/F_0$ = 6 (purified protein)	0.0041	9
eLACCO1.1	GFP	TTHA0766 (Ca^{2+}-dependent)	Intensiometric (extracellular)	$\Delta F/F_0$ = 4 (purified protein)	3.9	9
eLACCO2	GFP	TTHA0766 (Ca^{2+}-dependent)	Intensiometric (extracellular)	$\Delta F/F_0$ = 16 (purified protein)	0.28	10
eLACCO2.1	GFP	TTHA0766 (Ca^{2+}-dependent)	Intensiometric (extracellular)	$\Delta F/F_0$ = 14 (purified protein)	0.96	10
LiLac	mTurquoise 2	TlpC (extracellular dCACHE domain)	Lifetime (LT) (intracellular)	ΔLT = 1.2 ns (HEK293T cells)	2.7	11
***Discosoma* Red Fluorescent Protein (RFP)-based**						
R-iLACCO1	mApple	LldR (no DNA-binding domain)	Intensiometric (intracellular)	$\Delta F/F_0$ = 20 (purified protein)	0.074	10
R-iLACCO1.1	mApple	LldR (no DNA-binding domain)	Intensiometric (intracellular)	$\Delta F/F_0$ = 22 (purified protein)	0.23	10
R-iLACCO1.2	mApple	LldR (no DNA-binding domain)	Intensiometric (intracellular)	$\Delta F/F_0$ = 15 (purified protein)	0.35	10
R-eLACCO2	mApple	TTHA0766 (Ca^{2+}-dependent)	Intensiometric (intracellular)	$\Delta F/F_0$ = 20 (purified protein)	0.46	12
R-eLACCO2.1	mApple	TTHA0766 (Ca^{2+}-dependent)	Intensiometric (intracellular)	$\Delta F/F_0$ = 12 (purified protein)	5.7	12
**FRET-based**						
Laconic	mTFP & Venus FRET pair	LldR (with DNA-binding domain)	Emission Ratiometric (intracellular)	$\Delta R/R_0$ = 0.38 (HEK293 cells)	0.008 & 0.83 (biphasic)	14
$FILLac_{10N0C}$	mTFP & Venus FRET pair	LldR (with DNA-binding domain)	Emission Ratiometric (intracellular)	$\Delta R/R_0$ = 0.33 (purified protein)	0.006	14

SUPPLEMENTARY TEXT

Analysis and modeling of L-lactate oscillations in HeLa cells.

Analysis of L-lactate oscillations in HeLa cells. Fluorescence signals of iLACCO1.2 versus time were examined to characterize individual cell L-lactate oscillations following previous models[15–17] with some modifications. The fluorescence signals of iLACCO1.2 versus time in each cell were subjected to baseline subtraction to eliminate any long-term trends. Baselines were calculated as the walking average of the fluorescence data using a 100 s time window. Noise reduction was achieved by Fourier bandpass filtering with cutoff frequencies higher than 60.0 mHz and lower than 15.0 mHz.

The degree of synchronization in the entire population was characterized by the Kuramoto order parameter (R).[15–18] First, the Hilbert transform of the processed times series of cell i ($\underline{x_i}\ (t)$) was computed as follows:

$$\overline{x_i} = \frac{1}{\pi}\mathrm{PV}\int_{-\infty}^{\infty} \frac{x_i(t')}{t - t'}dt',$$

where PV is the Cauchy principal value of the integral, and $x_i(t')$ is the observed fluorescence signals from the biosensor. The instantaneous phase of each cell $\phi_i(t)$ was calculated as follows:

$$\phi_i(t) = \arctan\left(\frac{\overline{x_i(t)}}{x_i(t)}\right).$$

The instantaneous frequencies of each cell were calculated as the time derivatives of the unwrapped phases, followed by a smoothing by applying a sliding window of width 100 s.

The Kuramoto order parameter (R) was calculated as follows:

$$R(t) = \left|\frac{1}{N}\sum_{i}^{N} e^{i\phi_i(t)}\right|,$$

where N is the total cell number.

Mathematical model representing the glycolytic oscillations. To investigate the heterogeneity of the L-lactate oscillations, we examined a previously developed mathematical model[19] (**Figure 6C**), which is written as follows:

$$\frac{dG}{dt} = J_{\mathrm{GLUT}} - v_1,$$

$$\frac{dX}{dt} = v_1 - v_2,$$

$$\frac{dY}{dt} = 2v_2 - v_3 - J_{\mathrm{P,Y}},$$

$$\frac{dA_3}{dt} = -2v_1 + 4v_2 - v_4,$$

$$\frac{dG_{\mathrm{ex}}}{dt} = J_{\mathrm{in}} - \varphi J_{\mathrm{GLUT}},$$

$$\frac{dY_{\mathrm{ex}}}{dt} = \varphi J_{\mathrm{P,Y}}.$$

This model incorporates six variables; G, intracellular glucose; X, intermediates after phosphofructokinase (PFK) reaction; Y, intermediates after pyruvate kinase (PK) reaction including L-lactate; A_3, ATP; G_{ex}, extracellular glucose; Y_{ex}, extracellular Y. Total concentration of A_3 and A_2 (ADP) is assumed to be constant. The parameter φ represents the ratio of the cellular volume to the extracellular volume.

This model incorporates three transport processes; J_{in}, external input of glucose to the extracellular solution; J_{GLUT}, transport of the extracellular glucose into the cell through GLUT; $J_{\mathrm{P,Y}}$, membrane transport of triose including L-lactate. These transport processes are written as follows:

$$J_{\mathrm{in}} = \{\frac{G_i}{t_2 - t_1} (t_1 < t < t_2)\ 0\ (otherwise)\ ,$$

$$J_{\mathrm{GLUT}} = V_{\mathrm{max}} \frac{G_{\mathrm{ex}} - \frac{G}{K_{\mathrm{eq}}}}{K_{\mathrm{out}}\left(1 + \frac{G}{K_{\mathrm{in}}}\right) + G_{\mathrm{ex}}},$$

$$J_{\mathrm{P,Y}} = \kappa(Y - Y_{\mathrm{ex}}),$$

where V_{max} is the maximum velocity in the forward reaction; K_{out} is the affinity constants of the enzyme for extracellular glucose; K_{in} is the affinity constants of the enzyme for intracellular glucose; K_{eq} is the equilibrium constant; κ is a coupling constant, which is a function of the cell surface area, permeability of the membrane, and the cellular volume. In this study, G_{i}, t_1, and t_2 was set at 5 mM, 0 s, 1 s, respectively.

This model also incorporates four chemical reactions; v_1, PFK reaction that represents up-stream reactions in glycolysis; v_2, PK reaction that represents down-stream reactions in glycolysis; v_3, consumption reaction of the final product of glycolysis, including lactate; v_4, non-glycolytic ATP consumption. These reactions are written as follows:

$$v_1 = k_1 G A_3 \frac{{A_2}^m}{1 + {A_2}^m\left(\frac{1}{K_1} + \frac{G}{K_1 K_3} + \frac{A_3}{K_1 K_4}\right) + \frac{{A_3}^{m'}}{K_2}}$$

$$v_2 = k_2 X A_2 \frac{1}{1 + \frac{{A_3}^n}{K_5} + \frac{X}{K_6} + \frac{A_2}{K_7}},$$

$$v_3 = k_3 Y,$$

$$v_4 = k_4 A_3,$$

where K_1 and K_2 are dissociation constants of PFK-ATP and PFK-ADP complexes, respectively; K_3 and K_4 are dissociation constants for the subsequent enzyme-substrate complexes; K_5 is a dissociation constant of the PK-ATP complex; K_6 and K_7 are dissociation constants for the subsequent enzyme-substrate complexes; m is the number of PFK subunits; n is the number of PK subunits. The rate constants (k_1, k_2, k_3, k_4) are written as follows:

$$k_1 = 1.0 \times \alpha + 0.50,$$

$$k_2 = 0.50 \times \alpha + 0.0,$$

$$k_3 = 0.092 \times \alpha + 0.0082,$$

$$k_4 = 0.15 \times \alpha + 0.047.$$

The equations assume that the four rate constants can be different among the HeLa cells, but not totally random, as previously described.[19] The value of α is changed from 0.05 to 1 in increments of 0.05.

The values of the parameters were as follows:

$$V_{\mathrm{max}} = 0.65\ mM\ s^{-1}, K_{\mathrm{out}} = 10\ mM, K_{\mathrm{in}} = 12\ mM, K_{\mathrm{eq}} = 1, K_1 = 1\ mM^m, K_2 = 1\ mM^m, K_3 = 1\ mM,$$

$$K_4 = 1\ mM, K_5 = 20\ mM^n, K_6 = 20\ mM, K_7 = 20\ mM, \kappa = 0.01\ s^{-1}, \varphi = 0.01, m = n = 4.$$

The initial values of the variables were as follows:

$$G = 0.3, X = 0.3, Y = 0.3, A_3 = 0.3, A_2 = 2.7, G_{\mathrm{ex}} = 0, Y_{\mathrm{ex}} = 0.$$

These values are the same as in the previous study,[19] except for φ. The value for φ was changed because the extracellular volume can differ from the values in the previous study.[19] The effect of rate constant variation on the heterogeneity of the L-lactate oscillations was investigated by uniformly randomizing the values of V_{max}, k_1, k_2, k_3 and k_4 from 90% to 110% (**Figures 6D-E and S8G-H**). Additionally, the effect of variation in the initial concentrations of G, X, and Y were also investigated by generating these values from a Gaussian distribution with mean 0.3 and standard deviation 0.053 (**Figure S8I-L**). These variations are within the previously reported ranges.[19] Instantaneous frequencies of Y after glucose administration were investigated by simulating time courses for 1000 times with each α.

SUPPLEMENTARY REFERENCES

(1) Gao, Y.-G.; Suzuki, H.; Itou, H.; Zhou, Y.; Tanaka, Y.; Wachi, M.; Watanabe, N.; Tanaka, I.; Yao, M. Structural and Functional Characterization of the LldR from Corynebacterium Glutamicum: A Transcriptional Repressor Involved in L-Lactate and Sugar Utilization. *Nucleic Acids Res.* **2008**, *36* (22), 7110–7123.

(2) Fernandez-Fuentes, N.; Rai, B. K.; Madrid-Aliste, C. J.; Fajardo, J. E.; Fiser, A. Comparative Protein Structure Modeling by Combining Multiple Templates and Optimizing Sequence-to-Structure Alignments. *Bioinformatics* **2007**, *23* (19), 2558–2565.

(3) Jumper, J.; Evans, R.; Pritzel, A.; Green, T.; Figurnov, M.; Ronneberger, O.; Tunyasuvunakool, K.; Bates, R.; Žídek, A.; Potapenko, A.; et al. Highly Accurate Protein Structure Prediction with AlphaFold. *Nature* **2021**, *596* (7873), 583–589.

(4) Harada, K.; Chihara, T.; Hayasaka, Y.; Mita, M.; Takizawa, M.; Ishida, K.; Arai, M.; Tsuno, S.; Matsumoto, M.; Ishihara, T.; et al. Green Fluorescent Protein-Based Lactate and Pyruvate Indicators Suitable for Biochemical Assays and Live Cell Imaging. *Sci. Rep.* **2020**, *10* (1), 19562.

(5) Bekdash, R.; Quejada, J. R.; Ueno, S.; Kawano, F.; Morikawa, K.; Klein, A. D.; Matsumoto, K.; Lee, T. C.; Nakanishi, K.; Chalan, A.; et al. GEM-IL: A Highly Responsive Fluorescent Lactate Indicator. *Cell Rep Methods* **2021**, *1* (7), 100092.

(6) Aburto, C.; Galaz, A.; Bernier, A.; Sandoval, P. Y.; Holtheuer-Gallardo, S.; Ruminot, I.; Soto-Ojeda, I.; Hertenstein, H.; Schweizer, J. A.; Schirmeier, S.; et al. Single-Fluorophore Indicator to Explore Cellular and Sub-Cellular Lactate Dynamics. *ACS Sens* **2022**, *7* (11), 3278–3286.

(7) Li, X.; Zhang, Y.; Xu, L.; Wang, A.; Zou, Y.; Li, T.; Huang, L.; Chen, W.; Liu, S.; Jiang, K.; et al. Ultrasensitive Sensors Reveal the Spatiotemporal Landscape of Lactate Metabolism in Physiology and Disease. *Cell Metab.* **2023**, *35* (1), 200-211.e9.

(8) Wellbourne-Wood, J.; Briquet, M.; Alessandri, M.; Binda, F.; Touya, M.; Chatton, J.-Y. Evaluation of Hydroxycarboxylic Acid Receptor 1 (HCAR1) as a Building Block for Genetically Encoded Extracellular Lactate Biosensors. *Biosensors* **2022**, *12* (3), 143.

(9) Nasu, Y.; Murphy-Royal, C.; Wen, Y.; Haidey, J. N.; Molina, R. S.; Aggarwal, A.; Zhang, S.; Kamijo, Y.; Paquet, M.-E.; Podgorski, K.; et al. A Genetically Encoded Fluorescent Biosensor for Extracellular L-Lactate. *Nat. Commun.* **2021**, *12* (1), 7058.

(10) Nasu, Y.; Aggarwal, A.; Le, G. N. T.; Vo, C. T.; Kambe, Y.; Wang, X.; Beinlich, F. R. M.; Lee, A. B.; Ram, T. R.; Wang, F.; et al. Lactate Biosensors for Spectrally and Spatially Multiplexed Fluorescence Imaging. *Nat. Commun.* **2023**, *14* (1), 6598.

(11) Koveal, D.; Rosen, P. C.; Meyer, D. J.; Díaz-García, C. M.; Wang, Y.; Cai, L.-H.; Chou, P. J.; Weitz, D. A.; Yellen, G. A High-Throughput Multiparameter Screen for Accelerated Development and Optimization of Soluble Genetically Encoded Fluorescent Biosensors. *Nat.*

Commun. **2022**, *13* (1), 2919.

(12) Nasu, Y.; Kamijo, Y.; Hashizume, R.; Sato, H.; Hori, Y.; Tomita, T.; Drobizhev, M.; Campbell, R. E. A Red Fluorescent Genetically Encoded Biosensor for Extracellular L-Lactate. *bioRxiv* **2022**, 2022.08.30.505811.

(13) San Martín, A.; Ceballo, S.; Ruminot, I.; Lerchundi, R.; Frommer, W. B.; Barros, L. F. A Genetically Encoded FRET Lactate Sensor and Its Use to Detect the Warburg Effect in Single Cancer Cells. *PLoS One* **2013**, *8* (2), e57712.

(14) Xu, X.; Xu, R.; Hou, S.; Kang, Z.; Lü, C.; Wang, Q.; Zhang, W.; Wang, X.; Xu, P.; Gao, C.; et al. A Selective Fluorescent L-Lactate Biosensor Based on an l-Lactate-Specific Transcription Regulator and Förster Resonance Energy Transfer. *Biosensors* **2022**, *12* (12), 1111.

(15) Amemiya, T.; Shibata, K.; Itoh, Y.; Itoh, K.; Watanabe, M.; Yamaguchi, T. Primordial Oscillations in Life: Direct Observation of Glycolytic Oscillations in Individual HeLa Cervical Cancer Cells. *Chaos* **2017**, *27* (10), 104602.

(16) Weber, A.; Zuschratter, W.; Hauser, M. J. B. Partial Synchronisation of Glycolytic Oscillations in Yeast Cell Populations. *Sci. Rep.* **2020**, *10* (1), 19714.

(17) Mojica-Benavides, M.; van Niekerk, D. D.; Mijalkov, M.; Snoep, J. L.; Mehlig, B.; Volpe, G.; Goksör, M.; Adiels, C. B. Intercellular Communication Induces Glycolytic Synchronization Waves between Individually Oscillating Cells. *Proc. Natl. Acad. Sci. U. S. A.* **2021**, *118* (6), e2010075118.

(18) Kuramoto, Y. *Chemical Oscillations, Waves, and Turbulence*; Springer Berlin Heidelberg, 2011.

(19) Amemiya, T.; Shibata, K.; Du, Y.; Nakata, S.; Yamaguchi, T. Modeling Studies of Heterogeneities in Glycolytic Oscillations in HeLa Cervical Cancer Cells. *Chaos* **2019**, *29* (3), 033132.
